# Hematopoietic Stem Cell Gene Therapy for Brain Metastases Using Myeloid Cell–Specific Gene Promoters

**DOI:** 10.1093/jnci/djz181

**Published:** 2019-09-10

**Authors:** Tereza Andreou, Nora Rippaus, Krzysztof Wronski, Jennifer Williams, David Taggart, Stephanie Cherqui, Ashley Sunderland, Yolanda D Kartika, Teklu Egnuni, Rebecca J Brownlie, Ryan K Mathew, Sheri L Holmen, Christopher Fife, Alastair Droop, Mihaela Lorger

**Affiliations:** 1 School of Medicine, University of Leeds, Leeds, UK; 2 Department of Pediatrics, University of California San Diego, CA; 3 Leeds Institute for Data Analytics, University of Leeds, Leeds, UK; 4 Department of Neurosurgery, Leeds Teaching Hospitals NHS Trust, Leeds, UK; 5 Huntsman Cancer Institute, University of Utah, Salt Lake City, UT

## Abstract

**Background:**

Brain metastases (BrM) develop in 20–40% of cancer patients and represent an unmet clinical need. Limited access of drugs into the brain because of the blood-brain barrier is at least partially responsible for therapeutic failure, necessitating improved drug delivery systems.

**Methods:**

Green fluorescent protein (GFP)-transduced murine and nontransduced human hematopoietic stem cells (HSCs) were administered into mice (n = 10 and 3). The HSC progeny in mouse BrM and in patient-derived BrM tissue (n = 6) was characterized by flow cytometry and immunofluorescence. Promoters driving gene expression, specifically within the BrM-infiltrating HSC progeny, were identified through differential gene-expression analysis and subsequent validation of a series of promoter-green fluorescent protein-reporter constructs in mice (n = 5). One of the promoters was used to deliver tumor necrosis factor–related apoptosis-inducing ligand (TRAIL) to BrM in mice (n = 17/21 for TRAIL vs control group).

**Results:**

HSC progeny (consisting mostly of macrophages) efficiently homed to macrometastases (mean [SD] = 37.6% [7.2%] of all infiltrating cells for murine HSC progeny; 27.9% mean [SD] = 27.9% [4.9%] of infiltrating CD45+ hematopoietic cells for human HSC progeny) and micrometastases in mice (19.3–53.3% of all macrophages for murine HSCs). Macrophages were also abundant in patient-derived BrM tissue (mean [SD] = 8.8% [7.8%]). Collectively, this provided a rationale to optimize the delivery of gene therapy to BrM within myeloid cells. MMP14 promoter emerged as the strongest promoter construct capable of limiting gene expression to BrM-infiltrating myeloid cells in mice. TRAIL delivered under MMP14 promoter statistically significantly prolonged survival in mice (mean [SD] = 19.0 [3.4] vs mean [SD] = 15.0 [2.0] days for TRAIL vs control group; two-sided *P* = .006), demonstrating therapeutic and translational potential of our approach.

**Conclusions:**

Our study establishes HSC gene therapy using a myeloid cell–specific promoter as a new strategy to target BrM. This approach, with strong translational value, has potential to overcome the blood-brain barrier, target micrometastases, and control multifocal lesions.

Metastatic brain tumors are the most frequent intracranial tumors. They develop in 20–40% of all cancer patients, and mostly originate from lung cancer, breast cancer, and melanoma (1,2). The median survival time of patients with brain metastases (BrM) is only 4–19 months (1,3). Systemic chemotherapies have had little success in the treatment of BrM (1,2), which is thought to be at least partially because of the blood-brain barrier (BBB) limiting delivery of drugs into the brain (4,5). Despite partial disruption of the BBB in BrM, the vessel permeability in experimental BrM reaches only approximately 15% of that seen in other organs (4). Thus, novel approaches for the effective delivery of drugs to BrM are urgently required.

A handful of studies have explored neuronal and mesenchymal stem cells to deliver gene therapy to BrM in preclinical models (6–9), whereas hematopoietic stem cells (HSCs) have not yet been investigated in this context. We previously observed a substantial homing of macrophages, which are derived from HSCs, to BrM (10). Advantages of HSCs in comparison to other stem cell therapies include the ability to isolate them in large quantities and well-established procedures for their therapeutic use. Recent clinical trials of HSC gene therapy for Wiskott-Aldrich syndrome (11), X-linked severe combined immunodeficiency (12), β-thalassaemia (13), and adrenoleukodystrophy [a severe demyelinating brain disease (14,15)] showed remarkable results. The disadvantage of HSCs in the context of therapies, however, is the wide distribution of their progeny in different tissues, leading to systemic rather than localized delivery of transferred genes and thus potential systemic toxicities. To address this challenge, our goal was to develop a strategy for lentiviral gene transfer into HSCs that would restrict the delivery of transgenes to BrM.

## Methods

### In Vivo BrM Models

Six- to eight-week-old female C57Bl/6J and NSG mice were purchased from Charles River Laboratories, UK. Humanized NSG mice engrafted with human CD34+ cells used in immune cell quantification studies were purchased from JAX. Using stereotaxic apparatus (intracranial implantation model), we injected 1×10^5^ cancer cells into the striatum (16) or into the left internal carotid artery (10,17). Metacam (15 µg) was administered subcutaneously, and an inhalable anaesthetic (Isoflurane) was used during surgery. Bioluminescence imaging was performed using IVIS Spectrum (Perkin Elmer). All surgery and animal care procedures followed recommendations by the University of Leeds Animal Welfare & Ethical Review Committee and were performed under the approved UK Home Office project license in line with the Animal (Scientific Procedures) Act 1986.

### Bone Marrow Reconstitution

Bone marrow was flushed from the long bones of C57Bl/6J or C57Bl/6-Tg(Ubiquitin C [UBC]-green fluorescent protein [GFP])30Scha/J mice (Jackson Laboratories). HSCs were isolated using the Anti-Sca-1 Micro Bead Kit (Miltenyi) and were injected intravenously into lethally irradiated (8.45 Gy) C57Bl/6J mice either immediately (2.5×10^5^ cells) or following transduction with a respective lentiviral vector overnight (multiplicity of infection [MOI] 20–50; 1×10^6^ cells). Human CD34+ bone marrow–derived HSCs (DV Biologics) were transduced with pFUGW (18) (MOI 50) overnight and injected intravenously (2×10^5^ cells) into sublethally irradiated (225 cGy) NSG mice.

### In Vivo Survival Study

HSCs were lentivirally transduced with MMP14: GFP or MMP14: tumor necrosis factor–related apoptosis-inducing ligand (TRAIL) construct (MOI 20) and injected intravenously into lethally irradiated (8.45 Gy) C57Bl/6J mice (6-week-old females). Following bone marrow reconstitution (7 weeks later), 1×10^5^ PyMT cells were injected intracranially. Mice were examined daily for tumor growth–related symptoms, and symptoms were recorded according to the scoring sheet in our Home Office project license. At the onset of morbidity, the mice were culled, and brain tissue isolated for quantitative polymerase chain reaction (qPCR) analysis. Three mice were excluded from statistical analyses of the survival study: one mouse from each group because of non-tumor growth–related symptoms necessitating the animals be euthanized at an early time point and one mouse from the MMP14: GFP group because of failure to initiate tumor growth.

### Microarray Gene–Expression Analysis

Biological replicates used for analysis were as follows: n = 4 for PyMT BrM and the spleens of PyMT BrM-bearing mice; n = 1 for the bone marrow of PyMT BrM-bearing mice; n = 2 for EO771 BrM, naïve spleens, and naïve bone marrow. Toward the experimental endpoint, reduced size of spleens with black areas suggesting cell death was observed in mice with intracranial EO771 tumors. Consequently, we were unable to isolate viable spleen and bone marrow cells from this model despite repeating the experiment three times. Because of these difficulties, spleens and bone marrow isolated from naïve mice were used instead.

### Human Tissue

Human breast cancer BrM tissue and matched blood were obtained from the Leeds General Infirmary, the Leeds Teaching Hospitals Trust. Written informed consent was obtained from each individual under the ethical approval (Ref: 15/YH/0080) of the Leeds Multidisciplinary Research Tissue Bank or under research project ethical approval 18/EM/0159, approved by the Nottingham 1 Research Ethics Committee, UK.

### Statistical Analyses

Data were analyzed using a two-tailed *t* test or one-way analysis of variance with multiple comparisons, as stated in the figure legends (statistical significance cutoff = 0.05). Error bars represent SDs. Statistical significance in the survival study was determined via a two-sided log-rank test using GraphPad Prism 8.

Further methods are provided in the Supplementary Methods (available online).

## Results

### Interaction of Myeloid Cells with BrM

**Figure 1.**
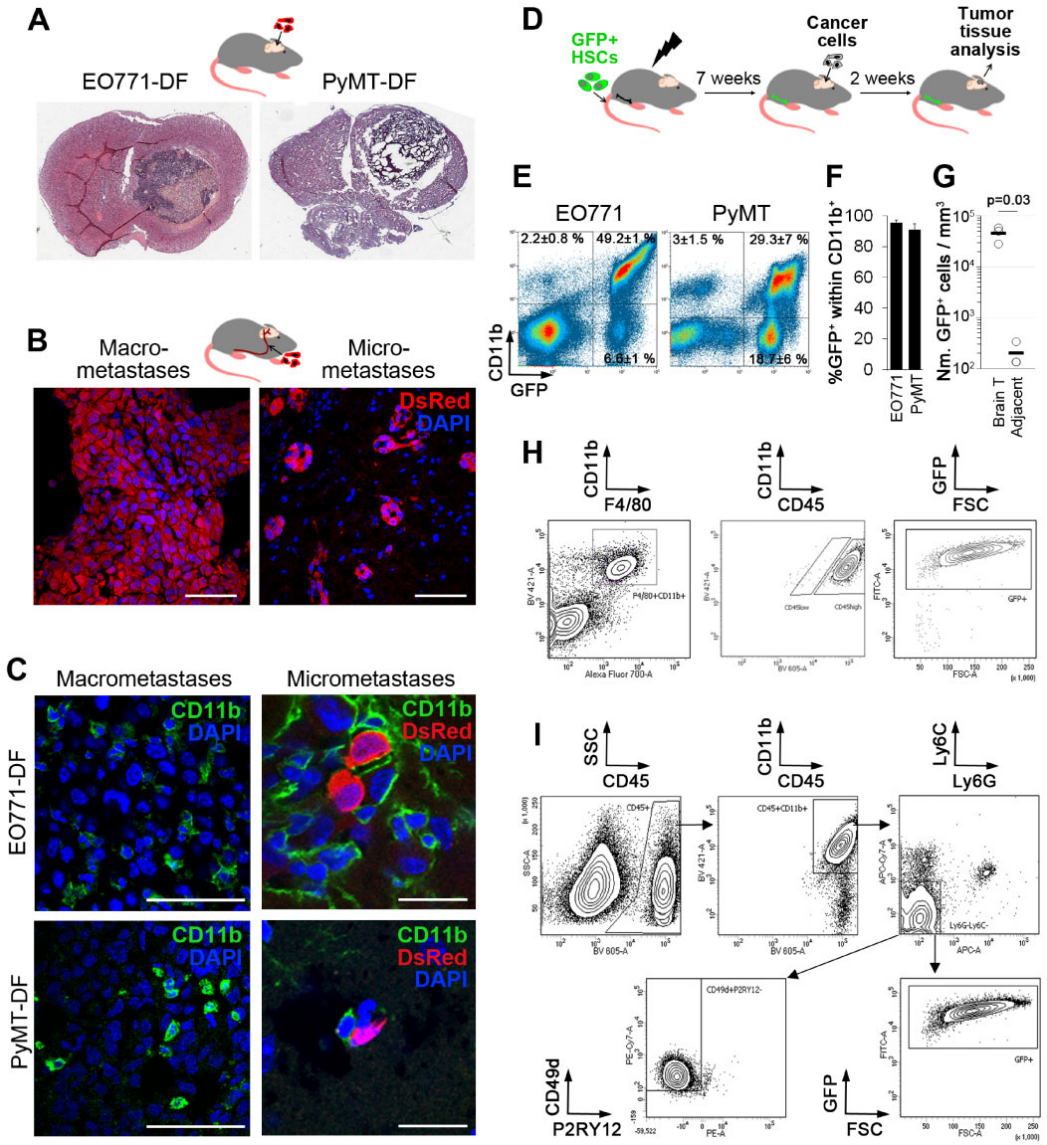
Myeloid cells in preclinical models of brain metastases. **A)** Discosoma sp. red fluorescent protein/Fluc (DF)–tagged EO771 and PyMT breast cancer cells give rise to a single large cancer lesion in the brain following intracranial implantation (H&E staining); n = 4. **B)** Administration of EO771-DF cancer cells into the internal carotid artery gives rise to micro- and macrometastases 2 weeks post-injection (n = 3/6 for EO771-DF/PyMT-DF). Scale bar = 50 µm. **C)** Macrometastases (intracranial implantation model) and micrometastases (carotid artery model) in the brain are infiltrated by CD11b+ myeloid cells (group sizes as in **A** and **B**). Scale bars = 50 µm (left panels) and 20 µm (right panels). **D–F)** Bone marrow in C57Bl/6J mice was ablated by irradiation, followed by transplantation of hematopoietic stem cells (HSCs) isolated from the transgenic mice expressing green fluorescent protein (GFP) downstream of the Ubiquitin C (UBC) promoter. Seven weeks later, tumors were generated by intracranial implantation of cancer cells. Infiltration of GFP+ bone marrow–derived myeloid cells (CD11b+) was quantified by flow cytometry at 2 weeks post–cancer cell injection (**E**). Percentage of GFP+ cells within the CD11b+ cell population is shown in (**F**); n = 5. **G)** Quantification of GFP+ HSC progeny per mm^3^ tissue within PyMT brain tumors and normal tumor-adjacent brain tissue (n = 3, *P* = .03. Statistically significant difference was determined with the two-tailed Student’s *t* test. **H)** Representative dot plots of flow cytometry for quantifying the percentage of CD45^high^ cells within CD11b+F4/80+ population and percentage of GFP+ cells within CD11b+F4/80+CD45^high^ cell population infiltrating PyMT tumors (n = 3). **I)** Gating strategy and quantification of GFP+ and CD49d+P2RY12- cells within CD45+CD11b+Ly6C-Ly6G- microglia/macrophages infiltrating PyMT tumors (n = 3).

To allow for in vivo detection, PyMT (10,19) and EO771 (20) murine breast cancer cell lines were tagged with Discosoma sp. red fluorescent protein and Firefly luciferase (EO771-DF and PyMT-DF). Both models formed large single lesions following intracranial implantation in C57Bl/6J mice (16) (Figure 1ASupplementary Figure 1A, available online). They also efficiently colonized the brain after administration into the internal carotid artery (10,17), resulting in multiple cancer lesions including micro- (few cells) and macrometastases (Figure 1B; Supplementary Figure 1B, available online), which grew in a close association with vasculature (Supplementary Figure 1C, available online). Thus, our models home to and colonize the brain, mimic multifocal metastatic lesions, and allow for the study of micro- and macrometastases, thereby recapitulating key aspects of the clinical disease.

As previously reported (10), CD11b+ myeloid cells were abundantly infiltrating large intracranial tumors and micrometastases in EO771-DF and PyMT-DF models (Figure 1C). Moreover, we detected substantial infiltration of F4/80+ microglia/macrophages within macrometastases in the brain in a spontaneous melanoma model (21) (Supplementary Figure 1F, available online). To determine the proportion of BrM-infiltrating cells originating from the HSCs as opposed to the yolk sack–derived brain-resident microglia (22), we generated bone marrow chimeras through transplantation of GFP+ HSCs into irradiated mice, resulting in mean (SD) = 73.4% (6.6%) GFP+ cells in the blood (Figure 1D; Supplementary Figure 1D, available online). The vast majority of CD11b+ cells within BrM were GFP+ (mean [SD] = 95.7% [1.5%] in EO771, mean [SD] = 90.9% [4.1%] in the PyMT model) and thus derived from the HSCs (Figure 1F). Strikingly, GFP+CD11b+ cells accounted for mean (SD) = 49.2% (1%) (EO771) and mean (SD) = 29.3% (7%) (PyMT) of all cells within BrM (Figure 1E). Infiltration of GFP+ HSC progeny into normal tumor-adjacent brain was statistically significantly lower than infiltration into tumors (mean [SD] = 4.5 × 10^4^ [1.5 × 10^4^] vs mean [SD] = 119.0 [114.0] cells per mm^3^ tissue within tumors vs tumor-adjacent brain; two-sided *P* = .03) (Figure 1G).

CD11b+F4/80+ macrophages/microglia represented mean (SD) = 32.7% (11.1%) (EO771) and mean (SD) = 12.5% (4.0%) (PyMT) of all intratumoral cells (Supplementary Figure 2A, available online). Whereas macrophages are generally CD45^high^ and microglia CD45^low^ (23), under specific conditions, microglia can also upregulate CD45 expression (24). Notably, mean (SD) = 98.5% (1.0%) of CD11b+F4/80+ cells (EO771 and PyMT tumors) were CD45^high^ (Figure 1H), and mean (SD) = 98.3% (0.7%) of F4/80+CD11b+CD45^high^ cells were GFP+ (Figure 1H; Supplementary Figure 2B, available online) and, therefore, derived from HSCs and not microglia. Additionally, we used recently reported macrophage [CD49d/ITGA4 (25)] and microglia-specific [P2RY12 (25–28)] cell surface markers. Within CD45+CD11b+Ly6G-Ly6C- macrophages/microglia, mean (SD) = 85.4% (6.7%) (EO771) and mean (SD) = 98.1% (1.3%) of cells (PyMT) were GFP+ (Figure 1I; Supplementary Figure 2C, available online), and mean (SD) = 82.2% (2.2%) (EO771) and mean (SD) = 96.1% (2.3%) of these cells (PyMT) were CD49d+P2RY12- (Figure 1I; Supplementary Figure 2D, available online), further confirming that the majority of myeloid cells are HSC-derived macrophages.

Within the F4/80+CD11b+ population infiltrating normal brain, mean (SD) = 66.6% (4.4%) of the cells were CD45^low^, and only mean (SD) = 8.9% (6.0%) within this population were HSC-derived GFP+ cells (Supplementary Figure 2E, available online). Interestingly, CD45^low^GFP+ cells retained low P2RY12 expression comparable to CD45^high^GFP+ macrophages (Supplementary Figure 2F, available online), suggesting they are distinct from brain-resident microglia with high P2RY12 expression, which is in line with other studies (28).

Notably, a proportion of myeloid cells in tumors could originate directly from a subpopulation of transplanted HSCs without hematopoiesis as shown previously for normal brain (28,29). Regardless, based on their capability to deliver transgenes to BrM, and based on their high proportion in intracranial tumors, we reasoned that HSC-derived myeloid cells could be used as cellular vehicles for the delivery of therapeutic genes to BrM.

### Homing of Genetically Modified HSC Progeny to BrM

**Figure 2.**
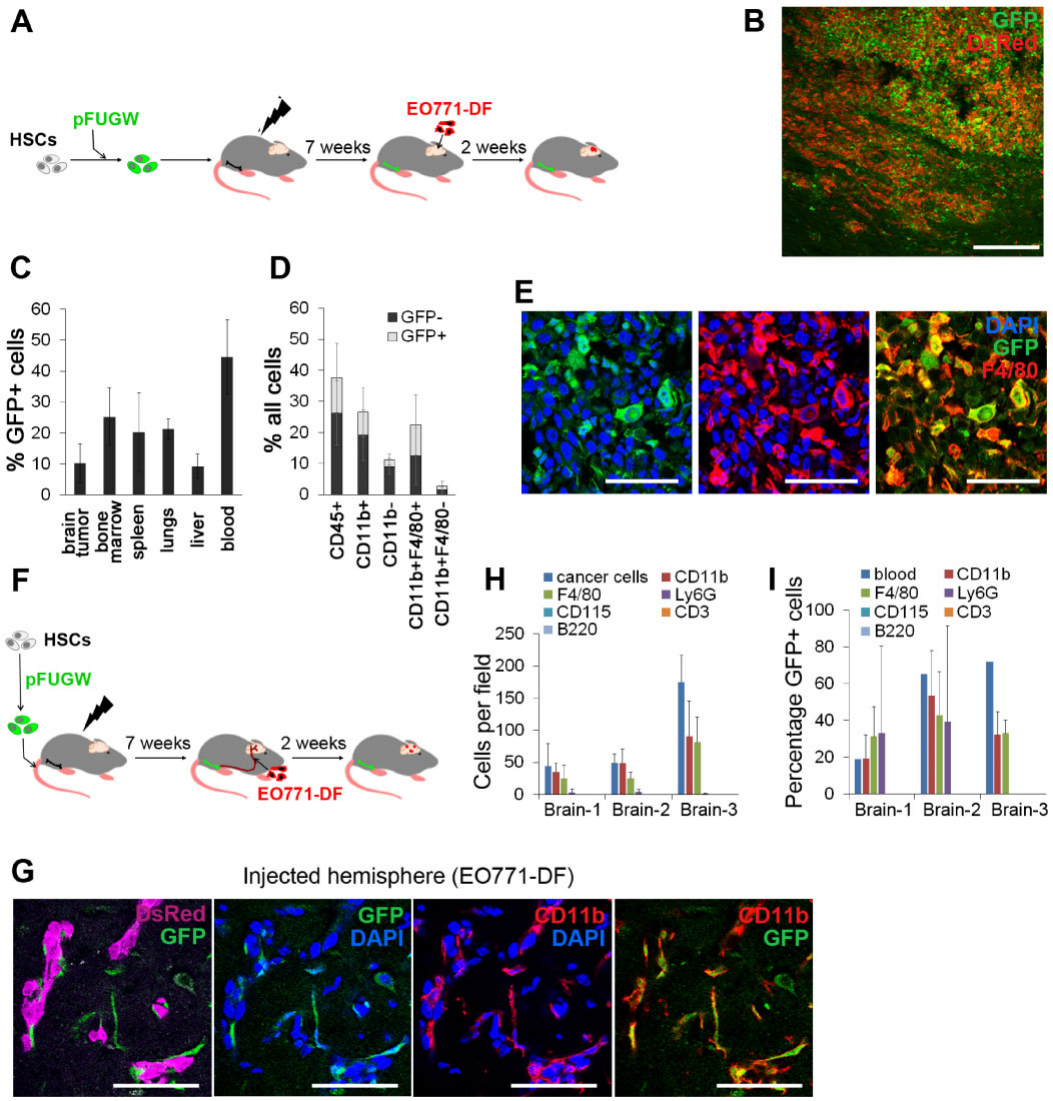
Genetically modified hematopoietic stem cell (HSC) progeny in the context of macro- and micrometastases in the brain. **A)** Murine HSCs transduced with lentiviral pFUGW vector were transplanted into lethally irradiated recipient mice, and tumors were generated by intracranial implantation of EO771-DF cells. **B)** Detection of pFUGW-transduced HSC progeny (green fluorescent protein [GFP]+) in intracranial tumors generated according to the scheme in **(A)** by immunofluorescence (n = 7). Scale bar = 200 µm. **C)** Quantification of pFUGW-transduced HSC progeny (GFP+) in different tissues by flow cytometry; n = 3. **D)** Quantification of immune cell populations within EO771 intracranial tumors by flow cytometry. Total percentage of individual cell populations, as well as the proportion of cells derived from pFUGW-transduced HSCs is shown; n = 7. **E)** Immunofluorescence staining of intracranial EO771 tumors for macrophages/microglia (F4/80+) and GFP (n = 4). Scale bars = 50 µm. **F)** Murine HSCs transduced with lentiviral pFUGW vector were transplanted into lethally irradiated recipient mice, followed by administration of EO771-DF cells into the internal carotid artery. **G)** CD11b+GFP+ progeny of HSCs was observed in close association with EO771-DF micrometastases in the brain. Fluorescence images were obtained by confocal microscopy (n = 3). Scale bars = 50 µm. **H)** Cancer cells and micrometastases-associated cells belonging to different hematopoietic cell subpopulations were counted on immunofluorescence images; 4–8 micrometastases-containing coronal brain sections per animal (n = 3) were quantified. **I)** Percentages of GFP+ cells within micrometastases-associated hematopoietic cell populations were quantified using immunofluorescence images and overall percentages of GFP+ cells in matched blood by flow cytometry (group sizes as in **H**).

To validate HSCs and their progeny as cellular vehicles, we used GFP as a model gene to be delivered to BrM. Murine HSCs were transduced with a lentiviral vector pFUGW, expressing GFP under the Ubiquitin C (UBC) promoter (18,30) and transplanted into lethally irradiated C57Bl/6J mice. BrM were generated by intracranial EO771-DF implantation (Figure 2A). The progeny of GFP-transduced HSCs efficiently homed to BrM (Figure 2B). As expected, GFP+ HSC progeny were also present in other organs to variable extent (Figure 2C).

In line with experiments using HSCs from GFP: UBC transgenic mice, the F4/80+ population contained almost exclusively macrophages (Supplementary Figure 1E, available online). CD45+ cells represented mean (SD) = 37.6% (7.2%) of all cells within tumors. The majority of CD45+ cells were CD11b+ myeloid cells (mean [SD] = 26.5% [6.0%] of all cells), consisting mostly of F4/80+ macrophages (mean [SD] = 22.4% [5.5%] of all cells) (Figure 2, D and E), whereas only mean (SD) = 2.8% (0.6%) of all cells belonged to other myeloid cell populations. The highest percentage of GFP+ cells (mean [SD] = 43.2% [38.6%]) was detected within macrophages. Notably, the infiltration of hematopoietic cells, including macrophages, into BrM remained unaltered following whole-body irradiation and HSC transplantation, as compared with the nonirradiated mice (Supplementary Figure 3A, available online).

The progeny of GFP-transduced HSCs also efficiently tracked down micrometastases and closely associated with small EO771 lesions (Figure 2, F and G). The majority of micrometastases-associated cells were CD11b+ and F4/80+ (Figure 2H), with 19.3–53.3% expressing GFP (Figure 2I). The ratio of cancer cells to CD11b+ cells (1:1 to 1:2) was similar to the intracranial model (Figure 2H). All micrometastases were associated with CD11b+ cells, and more than 90% contained CD11b+GFP+ cells (Supplementary Figure 3B, available online). Thus, we demonstrated that the genetically engineered HSC progeny, mostly consisting of macrophages, efficiently homed to large BrM and to micrometastases and can deliver genetically expressed molecules to the close proximity of cancer cells.

### Validation of Human HSCs and Their Progeny as Cellular Vehicles

**Figure 3.**
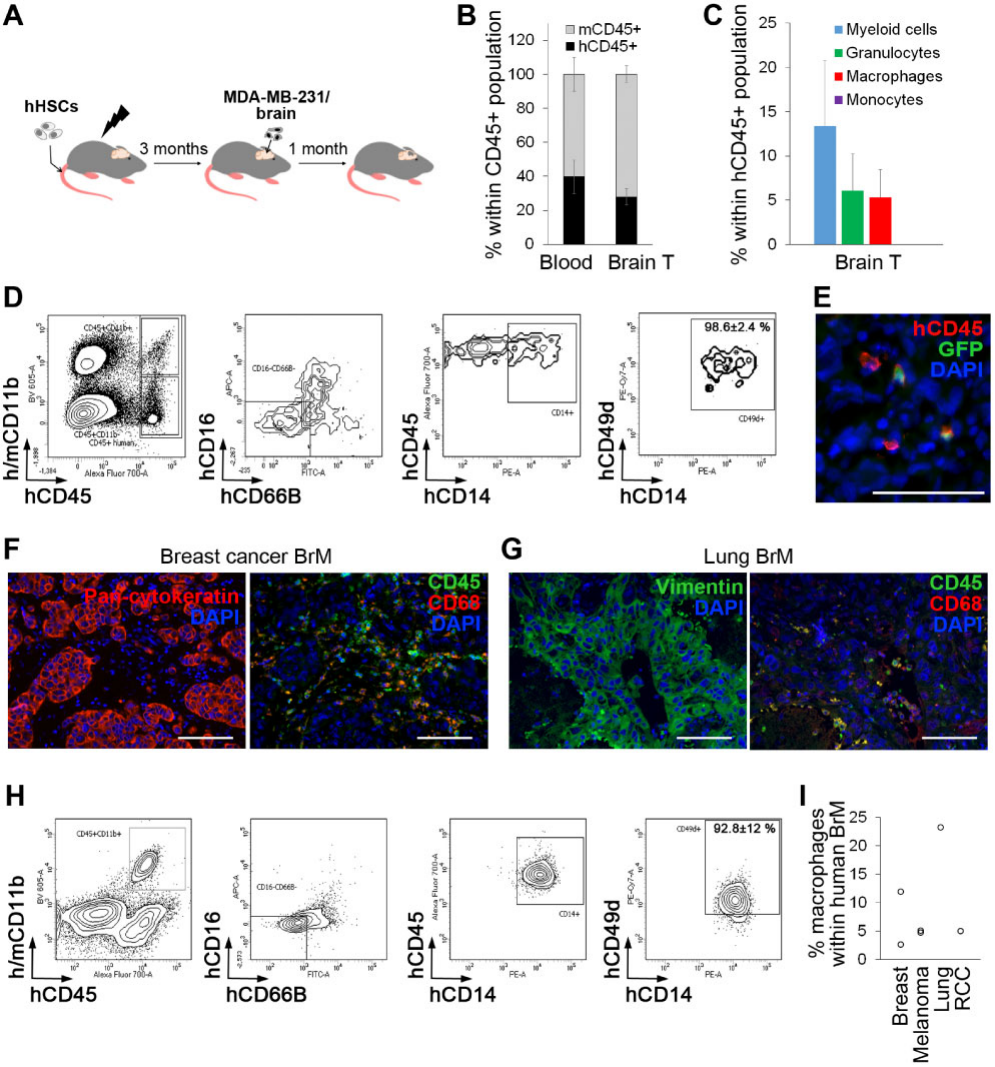
Human hematopoietic stem cells (HSCs) and their progeny as cellular vehicles targeting brain metastases. **A)** Tumors were generated in humanized NSG mice (JAX) 3–4 months after engraftment of human HSCs (hHSCs) by intracranial implantation of MDA-MB-231/brain cells. **B)** The percentage of human hematopoietic cells (hCD45+) in blood and brain tumors was quantified by flow cytometry (n = 3). **C)** Myeloid cell subpopulations within MDA-MB-231/brain tumors were quantified by flow cytometry (n = 3). **D)** Percentage of CD49d+ macrophages within human CD45+CD11b+CD16-CD66B-CD14+ population in intracranial MDA-MB-231/brain tumors was quantified by flow cytometry (n = 3). Representative dot plots are shown. **E)** Lentiviral pFUGW vector–transduced hHSCs were transplanted into sublethally irradiated NSG mice, and tumors were generated by intracranial implantation of MDA-MB-231/brain cells 3–4 months later (n = 4). Detection of green fluorescent protein-positive human hematopoietic cells in intracranial tumors was performed by immunofluorescence. Scale bar = 50 µm. **F, G)** Hematopoietic cells and macrophages in patient brain metastases specimens originating from breast cancer (**F**) and lung cancer (**G**) were detected by immunofluorescence (n = 3 per cancer type). To visualize cancer cells, adjacent sections were stained for pan-cytokeratin (breast cancer brain metastases [BrM]) or vimentin (lung cancer BrM) and costained for human CD45 (hematopoietic cells) human CD68 (macrophages). Scale bars = 100 µm. **H)** Percentage of CD49d+ macrophages within CD45+CD11b+CD16-CD66B-CD14+ microglia/macrophage population (25) in patient BrM originating from different primary cancer types as indicated in (**I**) was quantified by flow cytometry (n = 6). Representative dot plots are shown. **I)** Percentage of CD45+CD11b+CD16-CD66B-CD14+CD49d+ macrophages within total cell population in patient BrM (n = 6). RCC = renal cell carcinoma.

With clinical translation in mind, we next validated human HSCs (hHSCs). Engraftment of CD34+ hHSCs in sublethally irradiated NOD/SCID/IL2rγKO (NSG) mice, previously shown to result in mature and functional human hematopoietic cells (31), was followed by intracranial implantation of the human brain-homing breast cancer cell line MDA-MB-231/brain (32) (Figure 3A). Further, mean (SD) = 39.7% (9.9%) of hematopoietic cells in the blood and mean (SD) = 27.9% (4.9%) within tumors were of human origin (Figure 3B), with T and B cells being predominant in the blood, and T cells in brain tumors (Supplementary Figure 4A, available online).

Focusing on myeloid cells within tumors, this population (mean [SD] = 13.4% [7.4%] of human cells) consisted of granulocytes (mean [SD] =6.1% [4.2%]) and macrophages mean (SD) = (5.3% [3.1%]). In line with murine HSC models, mean (SD) = 98.6% (2.4%) of human CD45+CD11b+CD16-CD66B-CD14+ cells were CD49d+ macrophages (25) (Figure 3D). When transduced with pFUGW before transplantation into mice, hHSC progeny could also deliver GFP to brain tumors (Figure 3E; Supplementary Figure 4, B and C, available online).

Substantial infiltration of CD45+ hematopoietic cells and CD68+ macrophages/microglia was further detected in patient BrM originating from breast and lung cancer by immunofluorescence (Figure 3, F and G; Supplementary Figure 4, D, available online). Analysis of human BrM by flow cytometry revealed that the predominant population within CD45+CD11b+CD16-CD66B-CD14+ microglia/macrophages were CD49d+ macrophages (mean [SD] = 92.8% [12.1%]; n = 6) (Figure 3H; Supplementary Figure 4E, available online). Total percentage of macrophages within patient BrM varied between 2.7 and 23.3% (average of mean [SD] = 8.8% [7.8%]; n = 6) (Figure 3I). In summary, these data demonstrated strong parallels between human tissue and our preclinical models.

### Identification of Gene Promoters for BrM-Specific Delivery of Therapeutic Molecules within Myeloid Cells

**Figure 4.**
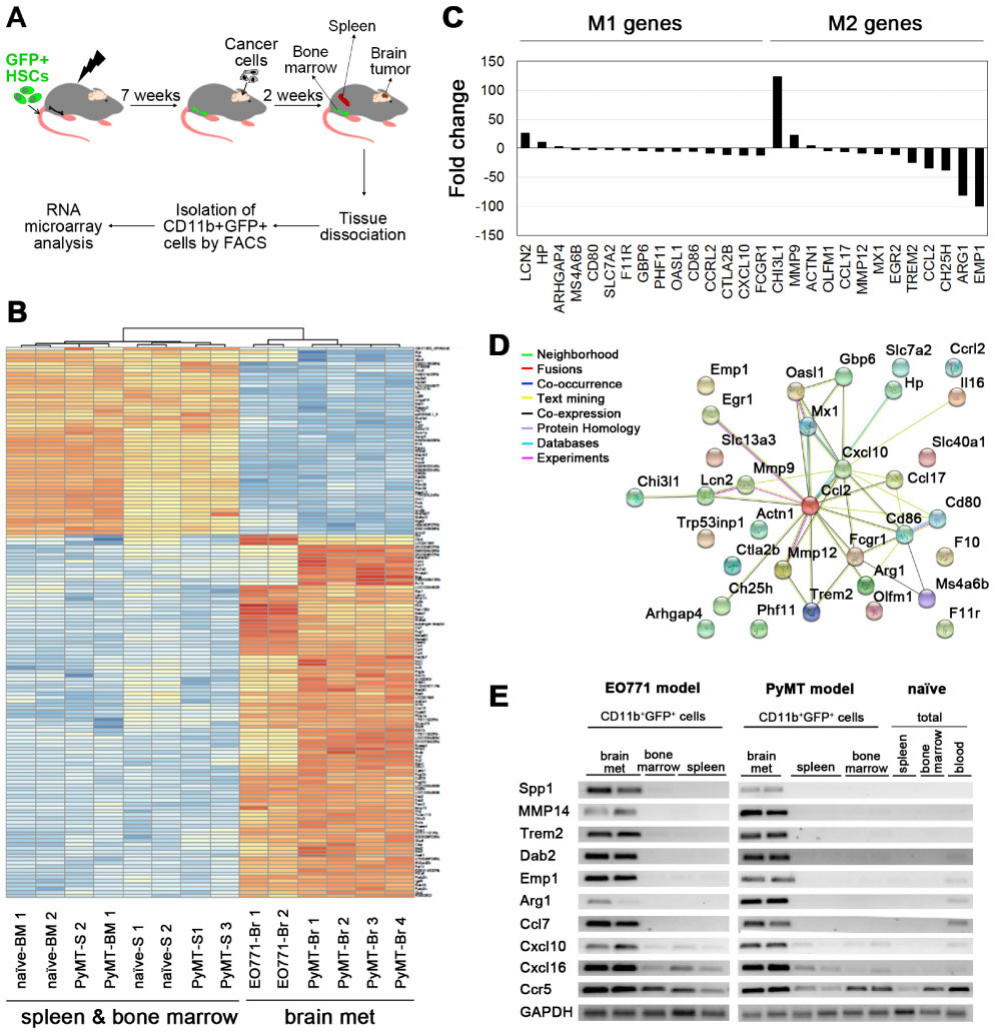
Identification of genes specific for brain metastases-infiltrating myeloid cells. **A)** Tumors in the brain were generated by intracranial implantation of cancer cells (untagged) into chimeric mice with green fluorescent protein (GFP)-tagged bone marrow (derived from Ubiquitin C [UBC]: GFP mice). CD11b+GFP+ cells were isolated from the brain tumors, spleens, and the bone marrow and subjected to RNA microarray analysis. Spleens and bone marrow isolated from naïve mice were used instead of those isolated from EO771 brain metastases (BrM)-bearing mice. **B)** A heat map of the top 150 probes differentially expressed between bone marrow-derived myeloid cells (CD11b+GFP+) isolated from BrM (Br) and myeloid cells isolated from the spleen (S) bone marrow (BM); n = 4 for PyMT BrM and the spleens; n = 1 for the PyMT bone marrow; n = 2 for EO771 BrM, naïve spleens, and naïve bone marrow. **C)** Differential expression of macrophage polarization–associated genes between BrM- and the spleen/bone marrow–derived myeloid cells. **D)** Functional protein interaction network of 33 murine macrophage polarization-associated genes, as identified using Search Tool for the Retrieval of Interacting Genes (STRING) (33). Individual nodes represent genes connected by color-coded lines of interaction according to software predictions (confidence score set to 0.4). **E)** Expression of top 10 genes upregulated in myeloid cells within brain metastases was validated by semiquantitative polymerase chain reaction. GADPH was used as a control. Representative DNA gels are shown. The quantification of the data is shown in Supplementary Figure S4B (available online). HSC = hematopoietic stem cell.

**Figure 5.**
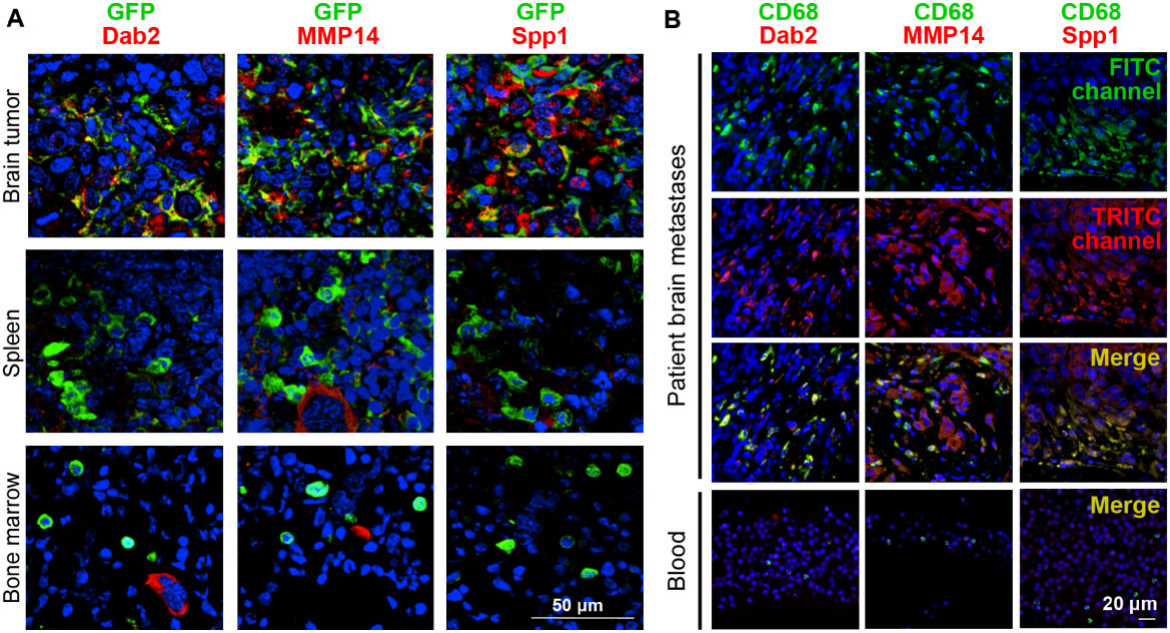
Validation of expression of genes specific for brain metastases (BrM)-infiltrating myeloid cells in preclinical models and clinical specimens. **A)** The activity of gene promoters in the progeny of pFUGW-transduced hematopoietic stem cells was assessed in BrM, the spleen, and bone marrow of EO771 tumor-bearing mice (intracranial implantation model) with chimeric green fluorescent protein (GFP)+ bone marrow by performing immunofluorescence staining for the respective proteins and GFP (n = 3). Colocalization of DAB2, MMP14, and SPP1, respectively, with GFP is shown in merge images. Nuclei are stained with DAPI. Scale bars = 50 µm. **B)** Human BrM tissue and donor-matched blood were costained for the macrophage marker CD68 and for DAB2, MMP14, and SPP1, respectively (n = 4). Three further brain metastases specimens with donor-matched blood samples are shown in Supplementary Figure S6 (available online). Nuclei are stained with DAPI. Scale bars = 20 µm.

To enable predominant delivery of gene therapy to BrM, we sought to identify gene promoters that are upregulated in BrM-infiltrating myeloid cells. GFP+CD11b+ cells were isolated from brain tumors, bone marrow, and the spleen of mice with chimeric GFP+ bone marrow (Figure 4A), bearing EO771 and PyMT tumors, respectively, or from naïve mice and subjected to a genome-wide gene-expression analysis. Data from both cancer models were initially combined, and BrM were compared with the pooled spleen/bone marrow group. Differential gene-expression analysis identified 5972 statistically significantly differentially expressed probes (False Discovery Rate [FDR] < 1%; Figure 4B), including chemokines, matrix metalloproteinases, and genes associated with macrophage activation/polarization (10,34,35). Expression of macrophage polarization markers (36–39) differed strongly between the intratumoral and the spleen/bone marrow–derived myeloid cells (Figure 4, C and D). Notably, 119 probes showed greater than 10-fold and 9 probes greater than 100-fold upregulation in BrM. However, only some of these probes were strongly upregulated in both cancer models. Expression of the latter (Supplementary Figure 5A, available online) was further validated by polymerase chain reaction (PCR) in CD11b+GFP+ and total cells isolated from different tissues (Figure 4E; Supplementary Figure 5B, available online). Among genes that showed the most specific expression in BrM-infiltrating CD11b+ cells were *Spp1*, *Mmp14*, *Trem2*, *Dab2*, and *Emp1*. Analysis in the EO771 model (mice transplanted with GFP-transduced HSCs) at the protein level revealed expression of DAB2, MMP14, and SPP1 in GFP+ BrM-infiltrating cells, but not in the spleen or bone marrow (Figure 5A; Supplementary Figure 6A, available online). DAB2, MMP14, and SPP1 were also consistently expressed in CD68+ microglia/macrophages in patient-derived breast cancer BrM (n = 4), while their expression was absent from the patient-matched blood (Figure 5B; Supplementary Figure 7, available online**)**. In summary, this suggested a strong potential of DAB2, MMP14, and SPP1 promoters to improve the specificity in the context of HSC gene therapy.

### Validation of BrM-Specific Myeloid Promoter Constructs in Preclinical Models

**Figure 6.**
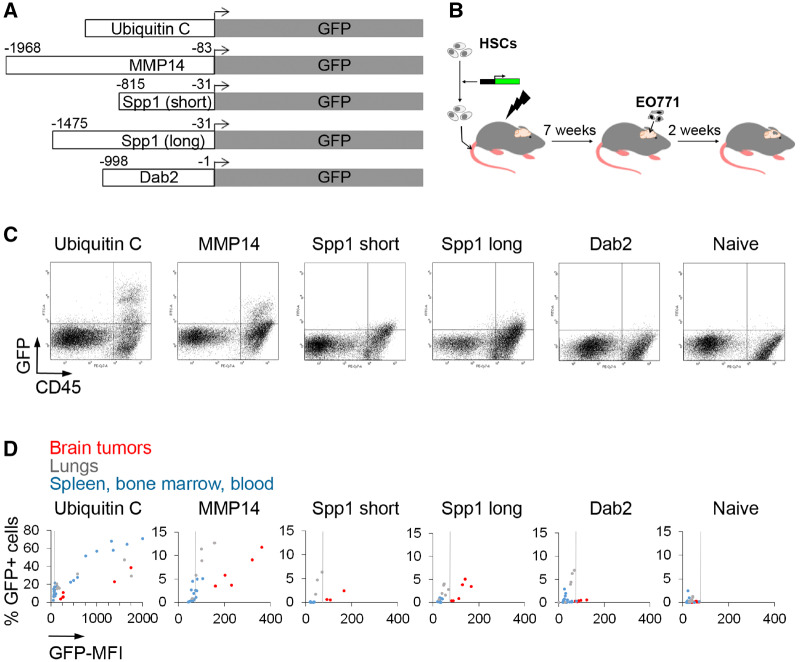
Validation of promoter constructs in preclinical models. **A)** Scheme of promoter-reporter constructs used for in vivo validation. The beginning and end of promoter fragments in relation to the transcriptional start site are indicated. **B)** Murine hematopoietic stem cells transduced with respective lentiviral promoter-reporter constructs (MOI 20) were transplanted into recipient mice, and tumors were generated by intracranial implantation of untagged EO771 cells. **C)** Representative flow cytometry dot plots showing green fluorescent protein (GFP) expression downstream of individual promoter constructs in EO771 brain metastases-infiltrating hematopoietic cells (n = 5). **D)** Percentage of GFP+ hematopoietic cells displayed against the GFP mean fluorescence intensity. Dots represent individual brain tumors, the lungs, and spleen/bone marrow/blood samples. The vertical line marks the background of GFP expression based on tissues isolated from the naïve mice (right). Individual values and statistical analysis are provided in Supplementary Tables 1 and 2 (available online) (n = 5).

Respective fragments of MMP14, SPP1 (two lengths), and DAB2 promoters were cloned into lentiviral vectors upstream of GFP (Figure 6A). HSCs transduced with these vectors were transplanted into lethally irradiated mice, followed by generation of intracranial EO771 tumors (Figure 6B). As expected, UBC promoter–driven GFP expression could be detected in CD45+ cells in all tissues without a tissue-specific pattern. By contrast, MMP14 and SPP1 promoters displayed statistically significantly higher GFP mean fluorescence intensity (MFI; a measure of promoter strength; see Supplementary Table 2, available online for *P* values) in CD45+ cells infiltrating BrM as compared with other tissues (Figure 6, C and D). Promoter strength (GFP MFI) was higher for the MMP14 promoter construct. To account for differences in the viral copy number (VCN) between different promoter constructs and tissues, we normalized MFI to VCN (MFI/VCN). As expected, VCN was higher in the bone marrow and spleen as compared with the brain tumors, whereas MFI/VCN was highest in the brain tumors. This confirmed an increased activity of MMP14 and SPP1 promoters in BrM-infiltrating vs spleen/bone marrow-infiltrating HSC progeny. In line with the MFI analysis, the MFI:VCN ratio was also the highest for the MMP14 promoter (Supplementary Figure 8A, available online). With the exception of the lungs, the percentage of GFP+ cells was higher in BrM than in other tissues for MMP14 and SPP1 promoter-reporter constructs (Figure 6D; Supplementary Table 1, available online). Based on these data, we focused on the MMP14 promoter.

Using mice that have received GFP+ HSCs from UBC: GFP transgenic mice, the expression of MMP14 in GFP+ cells in the brain was further analyzed by using immunofluorescence. GFP+ cells within normal brain were mainly located at the choroid plexus and ventricle walls, with lower numbers found within the brain parenchyma, cortex, cerebellum, and at the brain surface **(**Supplementary Figure 9, available online). In contrast to strong MMP14 expression in tumor-infiltrating GFP+ cells, GFP+ cells within normal brain were MMP14-negative (Supplementary Figure 9, available online). In line with this, analysis of previously published gene-expression data (25,27,28) revealed a statistically significantly higher expression of *Mmp14* in HSC-derived tumor-infiltrating macrophages vs HSC-derived normal brain-engrafting microglia-like cells (Supplementary Figure 8B, available online). In summary, this demonstrated an increased specificity of MMP14 expression in tumor-infiltrating hematopoietic cells.

Notably, CD45+ cells infiltrating BrM in a spontaneous melanoma model (21) also expressed MMP14 (Supplementary Figure 6B, available online), confirming that the MMP14 promoter is also active in BrM-infiltrating hematopoietic cells in a more physiological model and in the absence of irradiation.

### TRAIL Gene Therapy Delivered under the MMP14 Promoter

**Figure 7.**
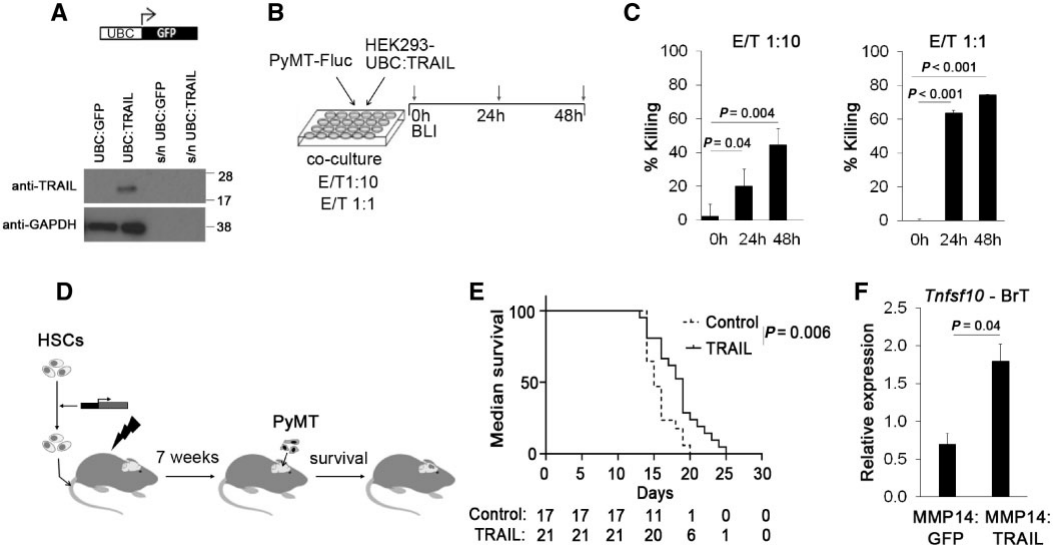
Delivery of lentiviral hematopoietic stem cell (HSC) therapy under the control of the MMP14 promoter fragment. **A)** Detection of murine tumor necrosis factor–related apoptosis-inducing ligand (TRAIL) in cell lysate and cell culture supernatant (s/n) of HEK293 cells transduced with Ubiquitin C (UBC): TRAIL construct. **B)** Schematic of experiment to confirm the sensitivity of PyMT cells to TRAIL in vitro. HEK293 UBC: TRAIL cells (effector cells; E) were cocultured with PyMT-Fluc cancer cells (target cells; T) at two different effector/target (E/T) ratios as indicated, and bioluminescence signal intensity (corresponding to the live PyMT cells) quantified after 24 and 48 hours. **C)** Percentage dead PyMT-Fluc cells (% killing) when cocultured with HEK293 UBC: TRAIL cells as quantified by bioluminescence imaging. Bioluminescence signals obtained in coculture of PyMT-Fluc cells with HEK293 UBC: TRAIL cells were normalized to the bioluminescence signals obtained in coculture with HEK293 UBC: green fluorescent protein (GFP) cells (n = 3; 2 independent experiments). Statistical analysis was performed using a two-tailed *t* test; *P* = .04 for 0 vs 24 and 48 hours at E/T 1: 10; *P* < .001 for 0 vs 24 and 48 hours at E/T 1: 1. **D)** Schematic of the survival experiment: HSCs were lentivirally transduced with MMP14: GFP or MMP14: TRAIL constructs, and injected into lethally irradiated mice. Following bone marrow reconstitution, PyMT cells were injected intracranially, and survival of the animals was monitored. **E)** Survival of mice reconstituted with lentivirally transduced MMP14: TRAIL HSCs compared with mice reconstituted with lentivirally transduced MMP14: GFP HSCs (19±3.4 vs 15±2.0 days, *P* = .006, two-sided log-rank test, n = 17/21 for MMP14: GFP/MMP14: TRAIL group, pooled data from two independent experiments). **F)** *Tnfsf10* (murine TRAIL gene) expression in brain tumor (BrT) tissue was detected by quantitative polymerase chain reaction. *Tnfsf10* Ct values were normalized to *Gapdh* Ct values using comparative Ct method (1.80 ± 0.23 vs 0.70 ± 0.14 for MMP14: TRAIL/MMP14: GFP group, *P* = .04, two-tailed *t* test, n = 7 per group).

Delivery of proapoptotic molecule TRAIL (9) was chosen as a proof of principle of the therapeutic applicability of our approach. After confirming the expression of the TRAIL construct under the UBC promoter (Figure 7A) and the sensitivity of PyMT cancer cells to the TRAIL in vitro (Figure 7, B and C), the TRAIL was placed under the MMP14 promoter fragment in a lentiviral vector and was used for in vivo efficacy study in the intracranial PyMT model (Figure 7D). Survival analysis revealed a statistically significantly prolonged survival of mice following TRAIL delivery in HSCs (mean [SD] = 19 [3.4] vs 15 [2.0] days for MMP14: TRAIL vs MMP14: GFP; two-sided *P* = .006) (Figure 7E), together with increased *Tnsf10* (TRAIL gene) expression in brain tumors as determined by qPCR (Figure 7F). In summary, our data establish a new platform for the delivery of therapeutic genes to BrM.

## Discussion

In this study, we developed a strategy for the delivery of therapeutic genes to BrM within the HSC progeny, using gene promoters specific for tumor-infiltrating myeloid cells. The latter were identified as the optimal cellular vehicles because of their high presence in BrM, their uniform distribution within tumors, and predominant homing to the tumor rather than healthy brain. Moreover, this strategy is expected to circumvent the BBB, control micrometastases, and enable a simultaneous targeting of multifocal BrM that pose a challenge for surgical removal. Use of myeloid cell–specific promoters is expected to limit the delivery of gene therapy mainly to tumors and thereby minimize systemic side effects. Whereas a similar approach using the Tie2-promoter has been previously used to deliver therapy to glioma in Tie2-expressing monocytes (40), here, we chose to focus on the most abundant hematopoietic cell population in BrM. In summary, our study establishes a novel approach for targeting of BrM with HSC gene therapy.

Lentiviral gene transfer demonstrated an excellent safety record (41,42), with promising results in patients (11–15). We demonstrated a strong potential for the clinical translation of HSC gene therapy for BrM by demonstrating the BrM-homing capacity of hHSCs, the abundant infiltration of myeloid cells, and the activity of identified myeloid promoters in human BrM. Because MMP14 is upregulated in brain-infiltrating macrophages in Alzheimer disease, multiple sclerosis, and stroke (43), this promoter could be also used for gene therapy in noncancerous brain disorders accompanied by strong myeloid cell infiltration (14,44–49). Moreover, our approach has the potential for simultaneous targeting of multiorgan metastases.

Several preclinical models were used in this study to address the key clinically relevant features of BrM, including the context of an intact immune system (syngeneic models), validation of human HSCs (humanized NSG mice), and analysis of macrometastases (intracranial model) and micrometastases (carotid artery model). Because approximately 15–60% of BrM remain undiagnosed (50,51), micrometastases in the brain may be quite common. Established BrM have also been shown to invade surrounding tissue (52,53). Thus, targeting of dormant micrometastases and those remaining after surgical removal (54,55) represents an unmet clinical need that could be addressed with our strategy.

In the present proof-of-principle study we used the MMP14 promoter. However, our study has several limitations. First, the initial differential gene-expression analysis was limited to the comparison of myeloid cells between brain tumors, bone marrow, and the spleen. Although we subsequently analyzed additional tissues, several tissues containing mature macrophages (ie, gut, liver, and fat) were not investigated because of technical limitations, and thus a possibility remains that our promoters are active in these tissues. Second, we reasoned that assessing the promoter strength at the protein level (flow cytometry) is superior, as proteins/peptides rather than mRNA represent effector molecules in therapeutic applications. However, use of an additional method (ie, qPCR quantification of transgene expression) would strengthen our findings. Third, we used whole-body irradiation before HSC transplantation. With clinical translation in mind, it will be important to determine in the future which HSC transplantation approach (ie, busulfan or treosulfan [29]) results in the highest engraftment of HSC progeny within tumors while minimizing engraftment within the healthy brain and other organs.

## Funding

The research leading to these results has received funding from the California Breast Cancer Research Program Innovative Developmental and Exploratory Award (IDEA) (to ML), the European Community’s Seventh Framework Programme (FP7/2007–2013) under grant agreement no. 294004 (to ML), Yorkshire Cancer Research award (to ML), the Brain Tumour Research and Support across Yorkshire award (to ML), CRUK Centre Leeds funding (to ML), the Biomedical and Health Research Centre award (to ML), and RO1CA121118 from the National Cancer Institute (to SLH). TA was supported by the Brain Tumour Charity; JW by Brain Tumour Research and Support across Yorkshire; and DT by Yorkshire Cancer Research. Tissue collection was partially supported by the PPR Foundation.

## Notes

The study funders had no role in the design of the study; the collection, analysis, or interpretation of the data; the writing of the article; or the decision to submit the article for publication. All authors declare no potential conflicts of interests.

We thank the cancer patients who donated their tissue. We thank Prof. Susan Short and the neuro-oncology multidisciplinary research team at the Leeds General Infirmary and St. James’s University Hospital in Leeds for help with tissue collection.

## Supplementary Material

djz181_Supplementary_Data
