# Supplementary material for: Hematopoietic Stem Cell Gene Therapy for Brain Metastases Using Myeloid Cell–Specific Gene Promoters
Source: J Natl Cancer Inst. 2019 Sep 10;112(6):617–27. doi: 10.1093/jnci/djz181 (PMC7301153; doi:10.1093/jnci/djz181)
Supplement: djz181_Supplementary_Data [file djz181_supplementary_data.pdf]

Supplementary methods

Cell lines

EO771 murine breast cancer cells [20] were kindly provided by Dr. Reisfeld from The Scripps Research Institute, La Jolla, CA. The cells were cultured in RPMI 1640 medium (Sigma, Dorset, UK) supplemented with 20% FBS (Labtech, East Sussex, UK), glutamine (Thermofisher, Paisley, UK), non-essential amino acids (Thermofisher, Paisley, UK), sodium pyruvate (Thermofisher, Paisley, UK), and penicillin/streptomycin (Sigma, Dorset, UK). PyMT 3503 cells [19] were derived from spontaneous mammary fat pad tumors in PyMT mice and were kindly provided by Dr. Ruf from The Scripps Research Institute, La Jolla, CA. The cells were grown in L-15 medium (Sigma, Dorset, UK) supplemented with 10% FBS, glutamine, and 10 ug/mL insulin (Sigma, Dorset, UK). MDA-MB-231/brain cells [32] were grown in EMEM medium (Sigma, Dorset, UK) supplemented with 10% FBS, glutamine, vitamin mix (Thermofisher, Paisley, UK), non-essential amino acids, and sodium pyruvate. The cells were stably transduced with Firefly luciferase and DsRed using lentiviral vectors pFUW-Fluc and pFUW-DsRed [16, 17]. HEK293 cells were obtained from ATCC and cultured in DMEM medium (Sigma, Dorset, UK) supplemented with 10% FBS, glutamine, and penicillin/streptomycin. HEK293Tcells were lentivirally transduced with MMP14:GFP or MMP14:TRAIL lentivirus to generate stable cell lines, HEK293-MMP14:GFP and HEK293-MMP14:TRAIL.

Tissue dissociation and flow cytometry

Mice blood was isolated from the tail vein and the lysis of red blood cells was performed prior to analysis using red cell lysis buffer (0.15M NH_4Cl, 10 mM $NaHCO_3$, 1 mM EDTA) for 4 minutes. Bone marrow cells were obtained by flushing the long bones. Splenocytes were isolated by mashing up the spleens. Brain metastases tissue (mouse and human), lungs, and liver were mechanically disrupted, followed by enzymatic dissociation with

collagenase (Roche; 3 mg/mL) and hyaluronidase (Sigma, Dorset, UK; 250 U/mL) for 20 minutes at 37°C. Subsequently the tissue was washed with cold facs incubation buffer (0.5% BSA, 2mM EDTA in PBS (Sigma, Dorset, UK)). For brain metastases, myelin was removed using Myelin removal beads (Miltenyi) according to the manufacturer's instructions. Dissociated cells were stained for different hematopoietic markers prior to their analysis.

Cells subsequently undergoing incubation with murine-specific antibodies (produced in rat) were blocked with 10% rat serum (Bio-Rad, Hertfordshire UK), and cells undergoing incubation with human-specific antibodies (produced in mouse) were blocked with 10% mouse serum (Bio-Rad, Hertfordshire UK) in facs incubation buffer for 10-30 minutes on ice. Fluorophore-tagged murine anti-CD45 (30-F11), anti-Ly6G (1A8), anti-ITGA4/CD49d (R1-2) and anti-P2RY12 (S16007D) were from Biolegend, anti-mouse/human CD11b (M1/70) and anti-Ly6C (AL-21) from BD Bioscience, anti-F4/80 (CI:A3-1) from AbD Serotec, and anti-CD3e (17A2) from eBioscience. Fluorophore-tagged human anti-CD45 (HI30), anti-CD33 (WM53), anti-CD66 (G10F5), anti-CD14 (HCD14), anti-CD16 (3G8) and anti-ITGA4/CD49d (9F10) were from Biolegend. The corresponding isotype control antibodies were from Biolegend, eBioscience or BD Bioscience. The incubation with antibodies was performed for 30-60 minutes on ice. Samples were analyzed on BD LSRII flow cytometer (BD Biosciences).

For the gene expression analysis, CD11b+GFP+ myeloid cells were isolated from a single-cell suspension obtained from collagenase/hyaluronidase-dissociated BrM tissue, spleens and the bone marrow by FACS using MoFlo Legacy (Beckman Coulter).
Flow cytometry data were quantified with Kaluza Flow analysis software from Beckman Coulter (Fig. 1E) or with FACSDiva software (all other data).

GFP mean fluorescence intensity (MFI) was quantified within CD45+ cell population isolated from different tissues in mice that have been reconstituted with promoter:reporter construct-transduced HSCs. To account for potential tissue-specific differences in background fluorescence, the MFI (FITC channel) of CD45+ cells isolated from

corresponding tissues from naïve mice was subtracted from the MFI of samples obtained from mice bearing promoter:reporter constructs.

Lentiviral packaging and concentration

HEK293T cells were co-transfected with the respective lentiviral expression plasmids and 3 packaging plasmids expressing Gag-pol, VSV-G and pRSV-Rev by calcium phosphate precipitation. Cell culture medium was changed the next morning. Virus-containing cell culture supernatant from transfected cells was collected at 24 and 48 hours post-medium change and concentrated (~300-fold) by centrifugation at *47.900 g* in a JA-18 rotor (Beckman Coulter) for 2.5 h at 4ºC. The virus was re-suspended in StemSpan SFEM medium (Stemcell Technologies) and stored at -80°C.

Determining lentiviral titers

Lentiviral titers were determined using the Lenti-X Provirus Quantitation kit (Clontech, CA, USA) and expressed as provirus copies/cell. Briefly, HEK293T cells were transduced with appropriate lentivirus, genomic DNA was isolated 96 h post-transduction using NucleoSpin Tissue kit (Macherey-Nagel) followed by qPCR amplification of provirus sequences in genomic DNA using TBGreen Advantage qPCR Premix (Takara, Saint-germain-en-laye, France) and the Standard curve method on a QuantStudio5 Real-Time PCR system (Applied Biosystems).

Lentiviral transduction of HSCs

During transduction, HSCs were cultured in retronectin-coated tissue culture plates in StemSpan SFEM medium (Stemcell Technologies). Murine SCF (100 ng/mL), TPO (100 ng/mL), FLT3 (100 ng/mL) and IL-3 (20 ng/mL) were added to murine HSCs, and human SCF (100 ng/mL), TPO (10 ng/mL), FLT-3 (100 ng/mL) and IL-3 (10 ng/mL) to human HSCs. The transduction was performed overnight.

Semi-quantitative RT-PCR

Total RNA was isolated using RNAqueous Total RNA Isolation Kit (Life Technologies). Single-strand cDNA was transcribed by Superscript III RT (Life Technologies) from 0.5 µg of RNA using oligo(dT)$_{20}$ primer (Life Technologies), following manufacturer's instructions. The relative expression level of glyceraldehyde-3-phosphate dehydrogenase (GAPDH) was used to normalize gene expression across different samples. PCR was performed with Pfu Ultra HF DNA polymerase (Agilent) using Veriti 96 well Thermal Cycler (Applied Biosystems). The following PCR program was used: denaturation step at 95°C for 2 minutes, annealing step for 30 seconds, and extension step at 72°C for 30 seconds. Sequences of gene-specific primer sets and annealing temperatures are given in Supplementary Table 3. PCR products were analyzed on a 2% agarose gel containing ethidium bromide. Data were quantified using ImageJ software and the intensity of individual bands across samples was normalized to the GAPDH control.

Generation of lentiviral constructs

For lentiviral promoter:reporter constructs, all promoter regions were amplified from mouse genomic DNA using PfuUltra High Fidelity DNA polymerase (Agilent) and appropriate primers as outlined below. The forward primers were designed to incorporate a PacI restriction site at the 5' end and the reverse primers were designed to incorporate an AgeI restriction site at the 3' end. Primers used to amplify promoter fragments by PCR, annealing temperatures, and information on specific promoter regions are summarized in Supplementary Table 4. Promoter regions were subcloned into pFUGW backbone [18] (PacI/AgeI restriction sites) using T4 DNA ligase kit (New England Biolabs). Ligation reactions were transformed into One shot Stbl3 bacterial cells (Thermofisher, Paisley, UK). This cloning resulted in a replacement of the Ubiquitin C promoter upstream of GFP with the respective MMP14, Spp1 and Dab2 promoter fragments. The resulting vectors were named pF-MMP14-GW, pF-SPP1short-GW, pF-SPP1long-GW and pF-DAB2-GW. The vectors

were propagated in the Stbl3 bacterial strain (Thermofisher, Paisley, UK) using appropriate antibiotics. All vector sequences were verified by DNA sequencing.

Plasmid DNA used in lentiviral packaging was isolated using S.N.A.P. plasmid DNA MidiKit (Life technologies).Murine TRAIL ORF was gene synthesized (Genscript) and subcloned into pFUGW backbone or pF-MMP14-GW using restriction digest (AgeI/EcoRI restriction sites), resulting in a replacement of the GFP gene with the *Tnfsf10* (murine TRAIL) gene.

Virus Copy Number analysis

Virus Copy Number (VCN) was determined with TBGreen Advantage qPCR Premix (Takara, Saint-germain-en-laye, France) using the standard curve method and primers binding to the GFP transgene (GFP1-fwd: 5'- GCCCATCCTGGTCGAGCT-3' and GFP2-rev: 5'-CTTGCCGTAGGTGGCATC-3', Applied Biosystems, 200 nM final concentration). The standard curves were generated using linearized (PacI, Thermofisher, Paisley, UK) and purified (using QIAquick PCR purification kit (Qiagen)) pFUGW plasmid as described previously [56]. The samples were PCR-amplified on a QuantStudio QS5 system (Applied Biosystems) using a two-step cycling protocol (40 cycles: 95°C for 5 sec, 62°C for 31 sec), preceded by a 30 sec incubation at 95°C. VCN values were normalized to the number of genomes in each sample.

Western Blot analysis

HEK293-MMP14:GFP and HEK293-MMP14:TRAIL cells were seeded in serum-containing medium and cultured for 24 hours. Medium was replaced for serum-free medium and the supernatant collected 48 hours later. Cell lysates were prepared using RIPA buffer (20 mM Tris pH 7.5, 100 mM NaCl, 1 mM EDTA, 0.5 mM DTT, 1% Triton-X-100, 0.1% SDS, 0.5% Deoxycholate), containing Complete mini protease inhibitor cocktail w/o EDTA (Roche, Hertfordshire, UK), 25 mM β-glycerophosphate, 1 mM Na_3VO_4 and 50 mM NaF. Cell lysates and cell culture supernatants were analyzed on denaturing 10 % polyacrylamide gel using

SDS/glycine protein running buffer, and transferred onto 0.45µM nitroceullulose membrane (Bio-Rad, Hertfordshire, UK) using SDS/glycine protein running buffer containing 20% methanol (Sigma, Dorset, UK). The membrane was blocked with blocking buffer (4% skimmed milk (Marvel), 1% BSA (Sigma, Dorset, UK)) for 1 hour at room temperature. Transferred proteins were detected with anti-TRAIL (GeneTex, GTX11700, 1:1000 dilution) and anti-GAPDH (Bio-Rad, VMA00046, 1:1000 dilution) primary antibodies in blocking buffer at 4°C overnight on a shaker, followed by detection with secondary anti-rabbit HRP (Invitrogen; G21234, 1:5000 dilution) and anti-mouse HRP antibody (Genetex, GTX213112-01, 1:5000 dilution) in blocking buffer for 1 hour at room temperature. ECL reagent (Thermofisher, Paisley, UK) was used to detect the signals following exposure of the membrane to high performance chemiluminescence film (GE healthcare, Chicago, US).

In vitro co-culture assays

Effector cells (HEK293-UBC:GFP or HEK293-UBC:TRAIL) were seeded together with target cells (PyMT-Fluc) in PyMT cell culture medium at two different effector to target cell ratios (E/T 1:10 and E/T 1:1). Cell viability was determined based on bioluminescence signal quantified using IVIS Spectrum (Perkin Elmer) upon addition of luciferin (3 ug/ml; Regis Technologies) at 0h, 24h and 48h. The bioluminescence signal of HEK293-UBC:TRAIL/PyMT-Fluc co-cultures was normalised to the signal of HEK293-UBC:GFP/PyMT/Fluc co-cultures.

RNA isolation from mouse tissue and cDNA synthesis

RNA from brain tumor tissue was isolated using the RNAqueous Micro-kit (Invitrogen), followed by DNaseI treatment (DNA-free DNA Removal kit; Ambion) to remove residual DNA. Agilent Tapestation System (Agilent) was used for RNA quality control. RNA concentration was determined with Agilent Tapestation System (Agilent) and ND-1000

spectrophotometer (Nanodrop®). cDNA synthesis was performed with SuperscriptIII reverse transcriptase (Invitrogen) at 65°C for 5 minutes for random priming, 50°C for 60 minutes for first strand synthesis, followed by a 15 minutes incubation at 70°C for enzyme inactivation.

Taqman qPCR assay

TRAIL expression in brain tumors was confirmed by qPCR using the Comparative Ct method and Taqman reagents (Taqman Universal Mastermix II, Applied Biosystems), using QuantStudio 5 (QS5) Real-Time PCR system (Applied Biosystems). The following Taqman probes were used: *Tnfsf10* (Mm01283603_m1), *Gapdh* (Mm99999915_g1) from ThermoFisher Scientific. The samples were amplified using a two-step cycling protocol (40 cycles: 95°C for 15 sec, 60°C for 1 min), subsequent to a 10 minutes incubation at 95°C.

Immunofluorescence analysis and tissue processing

Mice were perfused with saline, followed by perfusion with 4% paraformaldehyde (Sigma, Dorset, UK) in PBS, pH 7.5. The tissue was isolated, post-fixed in 4% paraformaldehyde overnight at 4°C, and incubated in 25% sucrose in sodium phosphate buffer for 72 hours at 4°C. Brain metastases tissue isolated from intracarotid models was cut into 40 μm free floating sections, which were stored in Walter's Antifreeze (sodium phosphate buffer, 30% Ethyleneglycol; 30% glycerol) at -20°C. Prior to staining, the floating sections were washed 3x with PBS. All other mouse tissue was embedded in OCT compound (VWR, Leicestershire, UK) and cut into 10 μm frozen sections on slides. Tissue was blocked with 10% goat serum (Thermofisher, Paisley, UK) / 0.03% Triton-X-100 (Sigma, Dorset, UK) in PBS for 1 hour at room temperature. Incubation with primary antibodies was performed at room temperature overnight. Anti-mouse/human-CD11b (M1/70) and anti-CD31 (MEC13.3) were from BD Bioscience, anti-mouse-F4/80 (CI:A3-1) from AbD Serotec, anti-human-CD45 (HI30), anti-human-CD68 (Y1/82A) and anti-human-HLA-DR (L243) were from Biolegend.

The polyclonal anti-GFP antibody was from Abcam, polyclonal anti-Dab2 and polyclonal anti-Trem2 were from Bioss, anti-MMP14 (EP1264Y) and anti-Spp1 (EPR3688) from GeneTex, and polyclonal anti-Emp1 from Abbiotec.

FFPE tissue from spontaneous melanoma brain metastases that developed in Dct::TVA;Braf^{CA};Cdkn2a$^{lox/lox}$;Pten$^{lox/lox}$ mice injected s.c. with viruses encoding myrAkt1 and Cre described in [21] was used in this study. No antigen retrieval (anti-F4/80 antibody CI:A3-1 from AbD Serotec) or heat mediated retrieval with EDTA, pH=8 (anti-CD45 HI30 from Biolegend; anti-MMP14 EP1264Y from GeneTex) was performed prior to blocking in 10% goat serum / 0.03% Triton-X-100 in PBS. Staining with the primary antibodies was performed overnight at room temperature.

Patient-derived tissue: whole blood cells after the lysis of red blood cells with RBC lysis buffer (Biolegend, London, UK) and brain metastases tissue were fixed in formalin for 48 hours and embedded in paraffin. Antigen heat retrieval with EDTA, pH=6 for 10 minutes was performed prior to blocking in 10% goat serum / 0.03% Triton-X-100 in PBS. Staining with the primary antibodies was performed overnight at room temperature. Anti-CD68 (PG-M1) was from DAKO, anti-human-CD45 (HI30) from Biolegend, anti-Pan-cytokeratin (C11) from Santa Cruz Biotechnology, anti-human Vimentin (Kl.9) was from DAKO, polyclonal anti-Dab2 from Bioss, anti-MMP14 (EP1264Y) and anti-Spp1 (EPR3688) from GeneTex.

The following secondary antibodies from Invitrogen were used at 1:500 dilution in blocking buffer: anti-mouse-Alexa488, anti-mouse-Alexa594, anti-rat-Alexa488, and anti-rabbit-Alexa488. The following secondary antibodies from Jackson ImmunoResearch were used at 1:200 dilution in blocking buffer: anti-rabbit-TRITC, anti-rat-Cy3, and anti-chicken-FITC. All incubations with secondary antibodies were performed for 1 hour at room temperature. Following the staining, the sections were stained with DAPI ((Sigma, Dorset, UK); 10 µg/mL) for 10 minutes at room temperature and mounted with Prolong Gold Antifade (Thermofisher, Paisley, UK). Images were acquired with EZ-C1 confocal microscope using EZ-C1 3.90 software (Nikon) or with AxioImager Z1 fluorescence microscope equipped with AxioCam MRc5 digital camera using AxioVision Rel. 4.7 software (Zeiss).

Microarray gene expression analysis

RNA was isolated using RNAqueous Micro Total RNA Isolation Kit (Ambion). The samples were processed using TotalPrep RNA amplification kit (Ambion) and hybridized to the MouseRef-8v2 WG-GX beadchips (Illumina). The arrays were processed according to the manufacturer's instructions.

Raw data were processed using GenomeStudio version 2011.1. Data processing and visualization were performed using R/Bioconductor. Microarray raw data pre-processing was performed using LIMMA[57]. Background correction was performed using the "normexp" algorithm[58] and normalization between arrays was performed using A-value quantile normalization[59]. Probes expressed in fewer than 3 arrays (using a detection p-value of < 0.05) were removed. Differential gene expression analysis between groups was performed using a linear model in LIMMA (FDR = 1%). Gene expression data was deposited in GEO, accession number GSE134026 (public access will be granted at the time point of publication).

Analysis of *Mmp14* expression in publicly available gene expression data sets

The accession numbers used to compare *Mmp14* gene expression in HSC-derived tumor-infiltrating macrophages versus HSC-derived normal brain-engrafting microglia-like cells were GEO: GSE86573, GSE122769 and GSE84819 [25, 27, 28]. Size factor normalization of raw counts within each dataset were carried out independently for each study using DESeq2 1.24.0 [60]. For comparison across studies, gene expression for each sample was normalized to that of a suitable housekeeping gene, *Polr2a*. All samples of interest were merged and z scores of gene expression were calculated across the concatenated cohort.

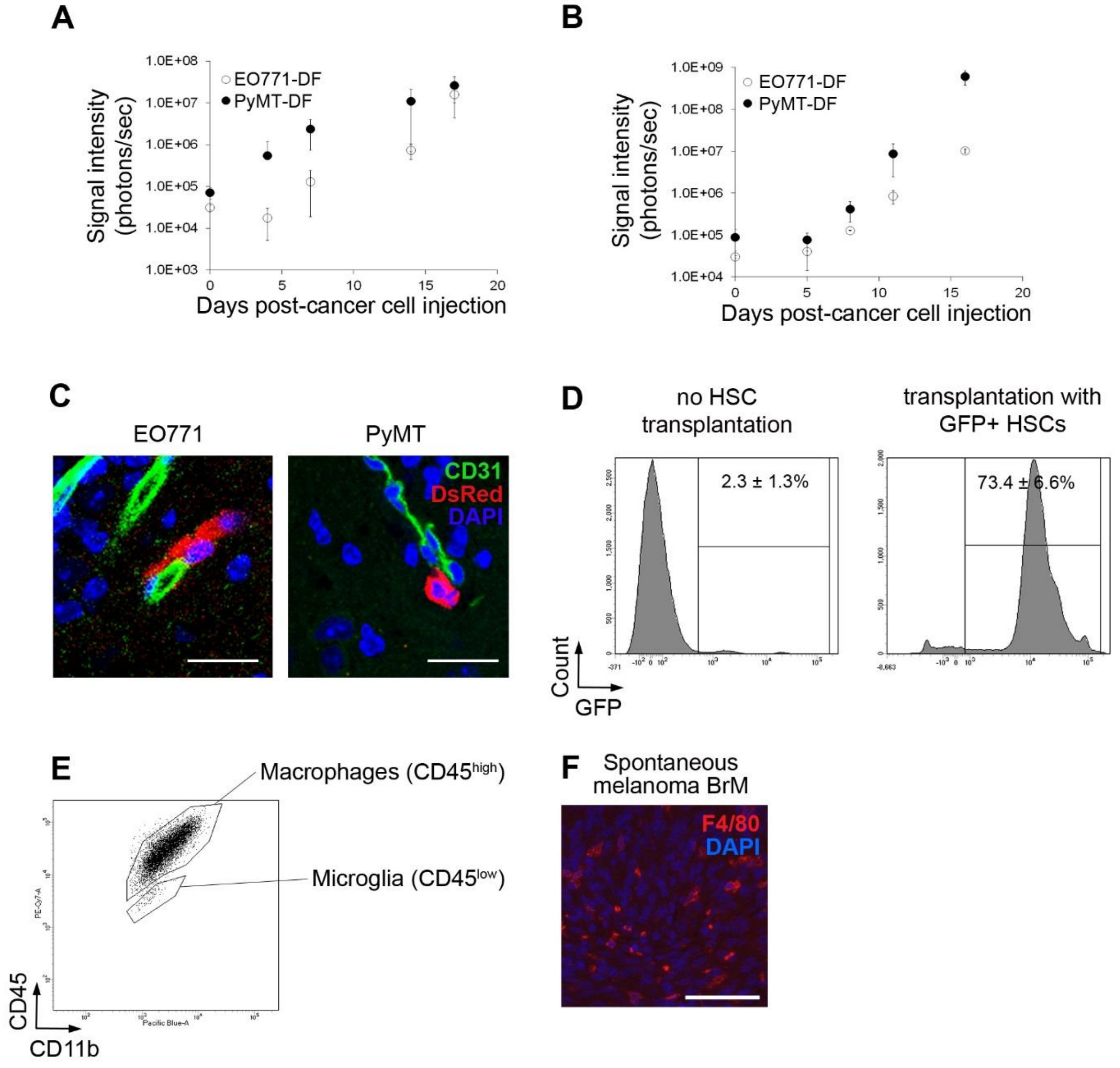

Supplementary Figure 1. Further characterization of BrM models. (A) Quantification of intracranial tumor growth by bioluminescence imaging, following intracranial cancer cell implantation; n=4. **(B)** Quantification of intracranial tumor growth by bioluminescence imaging, following administration of cancer cells into the internal carotid artery; n=3/6 for EO771-DF/PyMT-DF. **(C)** Following their administration into the internal carotid artery, cancer cells (DsRed+) were found in the brains in close association with blood vessels (CD31+). Scale bars = 20 µm. **(D)** Engraftment of murine GFP+ HSCs in C57Bl/6J mice was confirmed by quantifying GFP+ cells in mice blood by flow cytometry. The analysis was performed at 6 weeks post-bone marrow reconstitution. **(E)** Flow cytometry analysis of F4/80+ cells within EO771 BrM in intracranial implantation model. **(F)** Infiltration of F4/80+

microglia/macrophages in spontaneous melanoma brain metastases that developed in Dct::TVA;Braf^{CA};Cdkn2a$^{lox/lox}$;Pten$^{lox/lox}$ mice injected s.c. with viruses encoding myrAkt1 and Cre [21]. Scale bar = 50 µm.

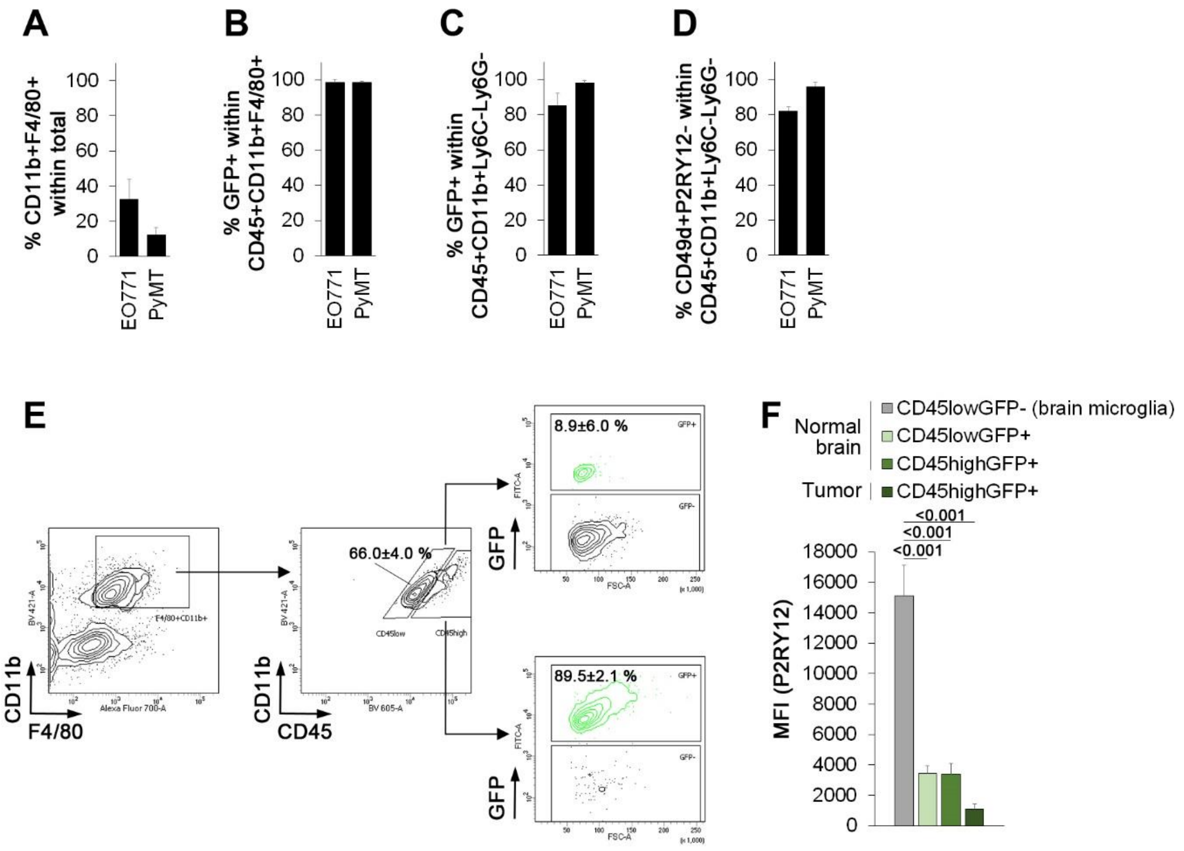

Supplementary Figure 2. Characterization of microglia/macrophages within tumors and adjacent normal brain tissue. (A) Quantification of CD11b+F4/80+ cells within total cell population within tumors by flow cytometry (n=3). **(B)** Quantification of GFP+ cells within CD45+CD11b+F4/80^{high} cell population within tumors by flow cytometry (n=3). **(C)** Quantification of GFP+ cells within CD45+CD11b+Ly6C-Ly6G- cell population within tumors by flow cytometry (n=3). **(D)** Quantification of CD49d+P2RY12- cells within CD45+CD11b+Ly6C-Ly6G- cell population within tumors by flow cytometry (n=3). **(E)** Representative dot plots and quantification of CD45^{high}/CD45^{low} and GFP+ cells within CD11b+F4/80+ microglia/macrophages in the normal tumor-adjacent brain (n=3). **(F)** Quantification of P2RY12 microglial marker expression levels (mean fluorescence intensity; MFI) in different CD11b+F4/80+ cell populations within normal tumor-adjacent brain and PyMT tumors by flow cytometry (n=3). Statistical differences were determined by one-way ANOVA with multiple comparisons.

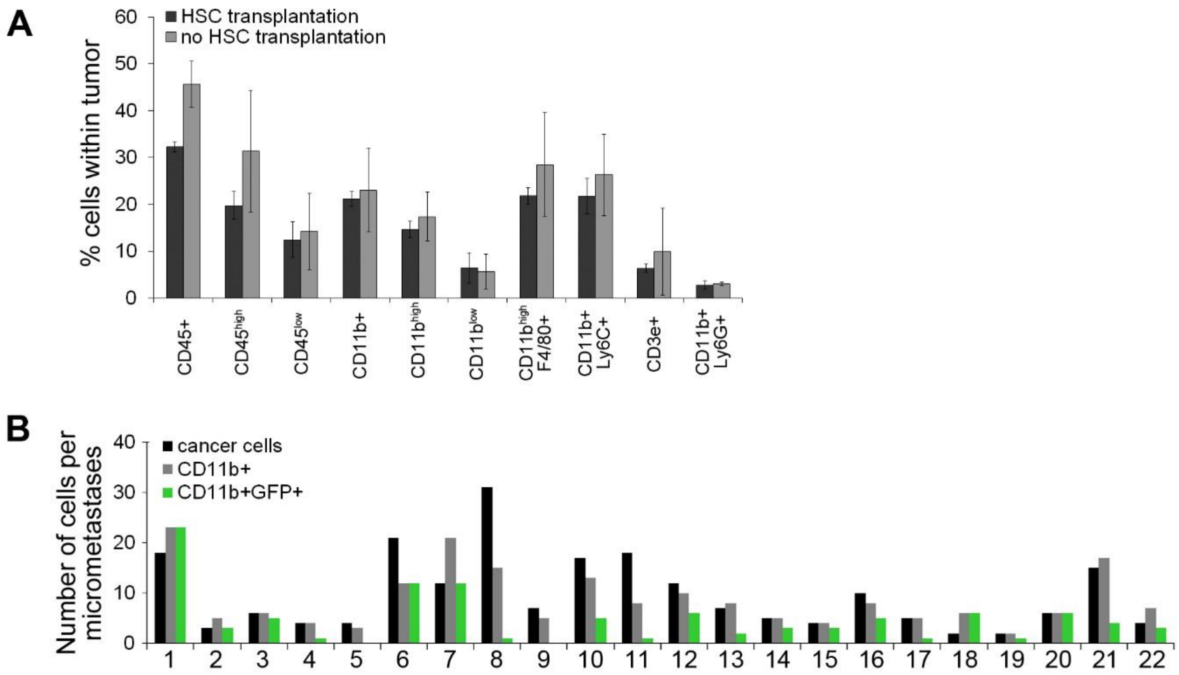

Supplementary Figure 3. Infiltration of HSC progeny into brain metastases. (A) The percentages of different brain metastases-infiltrating cell populations in mice that have received GFP-transduced HSCs upon bone marrow ablation (n=3; black bars) and in wild type mice that did not undergo bone marrow ablation or HSCs transplantation (n=2; grey bars) were quantified by FACS. **(B)** Numbers of cancer cells, CD11b+ and CD11b+GFP+ cells per individual micrometastases were quantified using immunofluorescence images (n=22).

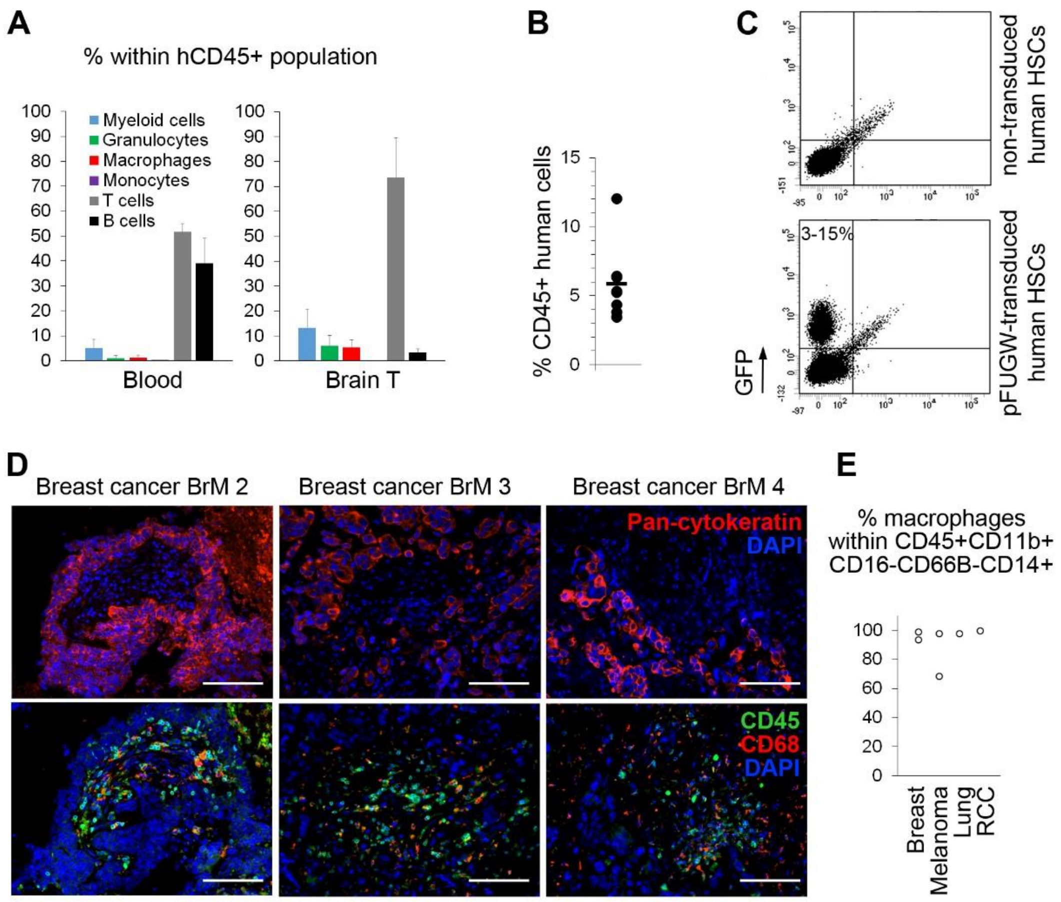

Supplementary Figure 4. Engraftment of human HSCs in NSG mice. (A) Human hematopoietic cell subpopulations were quantified in the blood (left) and brain tumors (right) of NSG mice transplanted with hHSCs at 3-4 months post-transplantation by flow cytometry (n=3). **(B)** The presence of human CD45+ hematopoietic cells in the blood of NSG mice was quantified by flow cytometry at the experimental endpoint (n=8). Four of the mice were transplanted with hHSCs transduced with pFUGW. **(C)** Detection of GFP+ cells in the bone marrow of NSG mice that received GFP-transduced human HSCs (right) in comparison to the mice that received non-transduced HSCs (left). **(D)** Infiltration of human brain metastases by hematopoietic cells and macrophages was detected by fluorescence microscopy in different specimens. Adjacent sections were stained for pan-cytokeratin (cancer cells) and co-stained for human CD45 (hematopoietic cells) / human CD68 (macrophages). Scale bars = 100 µm. **(E)** Percentage of CD49d+ macrophages within CD45+CD11b+CD16-CD66B-CD14+ cell population in patient BrM (n=6). The primary tumor origin of BrM is indicated.

A

	combined	EO771 model	PyMT model
**Ccl7**	258	180	148
**Arg1**	217	86	549
**Dab2**	119	130	118
**Emp1**	100	246	40
**Spp1**	89	96	82
**Ccr5**	73	100	93
**MMP14**	70	63	78
**Cxcl10**	51	158	7.5
**Cxcl16**	26	14	34
**Trem2**	25	29	21

B

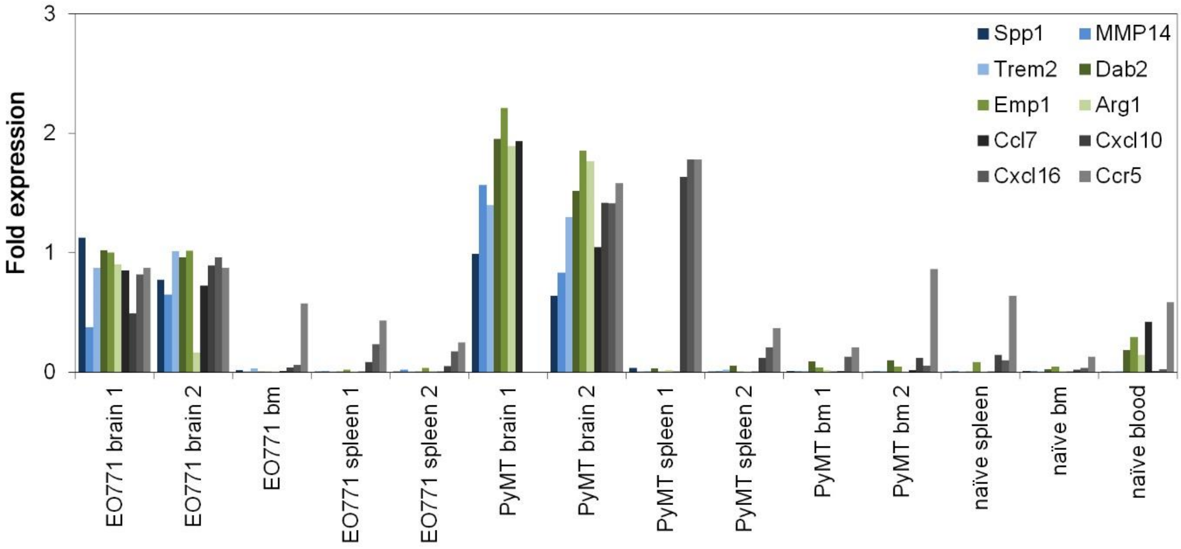

Supplementary Figure 5. Validation of genes up-regulated in brain metastases-infiltrating myeloid cells. (A) Fold changes in gene expression levels (up-regulation in brain metastases versus the spleen/bone marrow) for the 10 genes subjected to further validation are shown. The "combined" column is showing fold changes in expression levels obtained by comparing all brain metastases samples versus all spleen and bone marrow samples. The subsequent columns ("EO771 model" and "PyMT model") are showing fold changes obtained by comparing brain metastases samples from each individual cancer model versus the corresponding spleen and bone marrow samples. **(B)** Quantification of the gene expression data obtained by a semi-quantitative RT-PCR (a representative DNA gel is shown in Figure 4C). The intensity of individual bands was normalized to the intensity of *Gapdh*; bm = bone marrow;

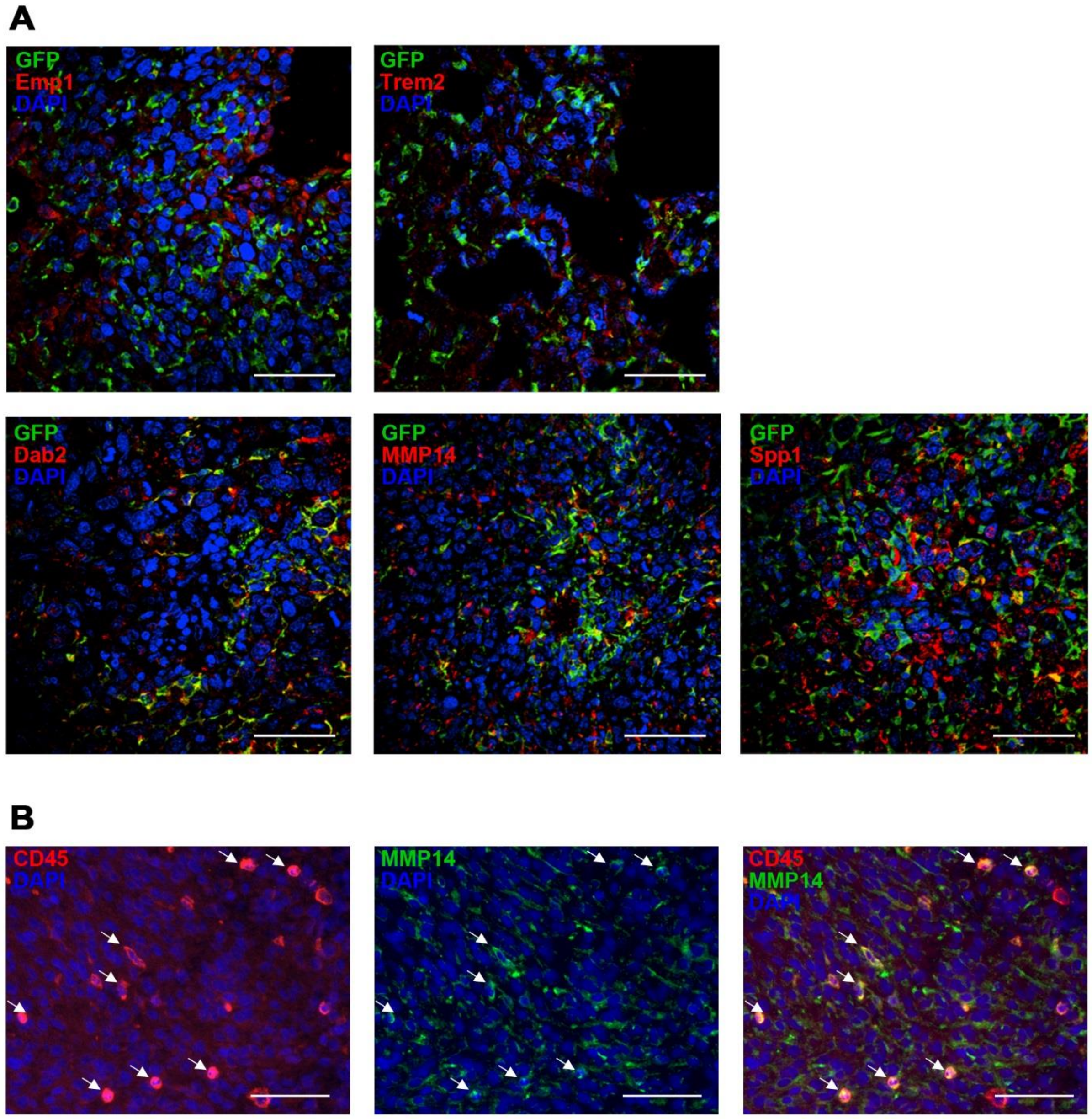

Supplementary Figure 6. Validation of myeloid cell promoters in preclinical models. **(A)** The activity of gene promoters in the progeny of pFUGW-transduced HSCs was assessed in brain metastases isolated from EO771-DF tumor-bearing mice with chimeric GFP+ bone marrow, by performing immunofluorescence staining for the respective proteins and GFP. Scale bars = 50 µm. **(B)** Expression of MMP14 in CD45+ cells infiltrating spontaneous melanoma brain metastases that developed in Dct::TVA;Braf^{CA};Cdkn2a$^{lox/lox}$;Pten$^{lox/lox}$ mice injected s.c. with viruses encoding myrAkt1 and Cre [21]. Scale bars = 50 µm.

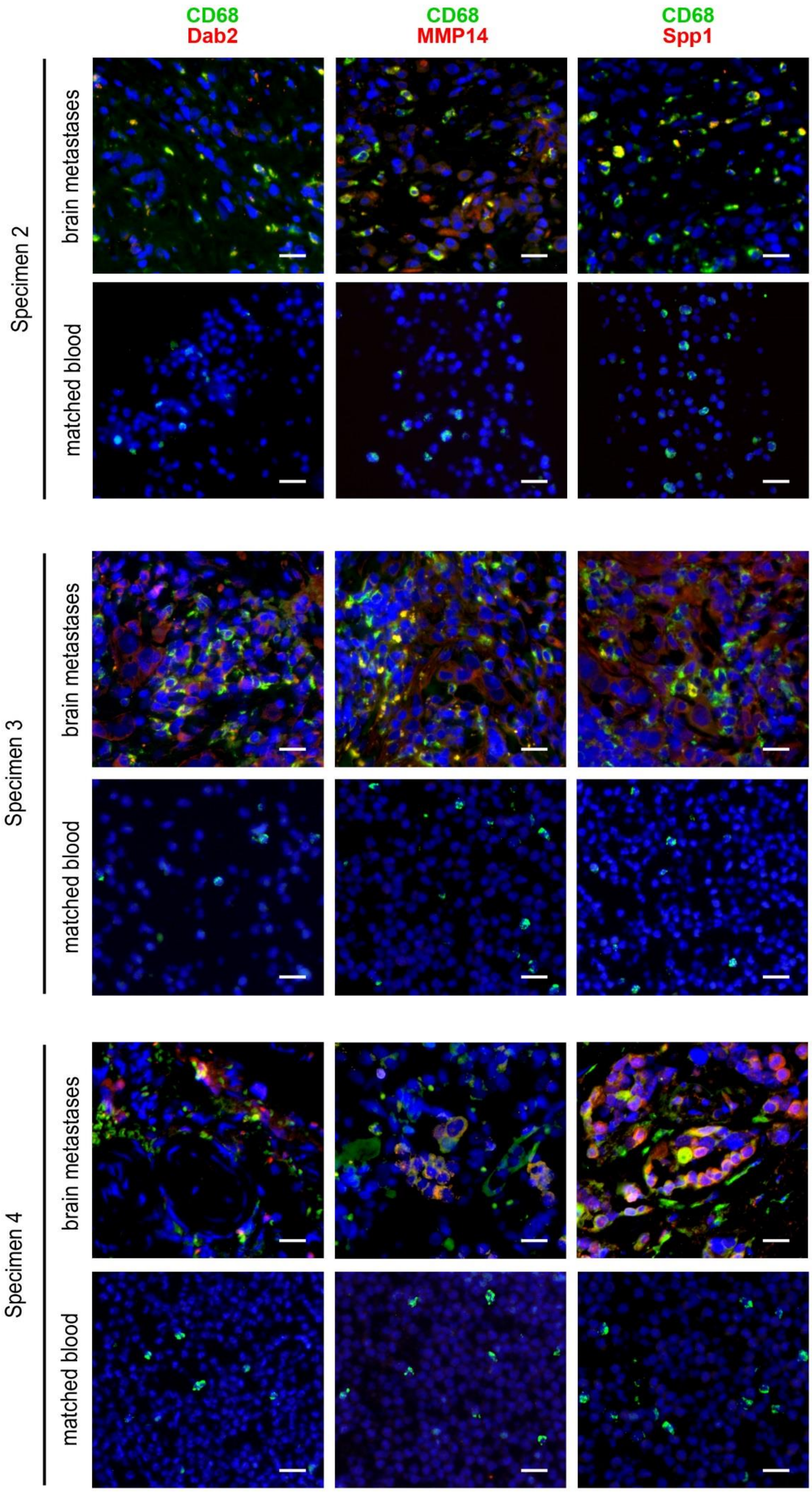
CD68
Dab2
CD68
MMP14
CD68
Spp1
Specimen 2
brain metastases
matched blood
Specimen 3
brain metastases
matched blood
Specimen 4
brain metastases
matched blood

Supplementary Figure 7. DAB2, MMP14 and SPP1 gene promoters demonstrate specific activity in brain metastases-infiltrating macrophages in human patients. Human brain metastases tissue and donor-matched blood were co-stained for the macrophage marker CD68 and for DAB2, MMP14 or SPP1. Nuclear staining (DAPI) is shown in blue. Scale bars = 20 µm.

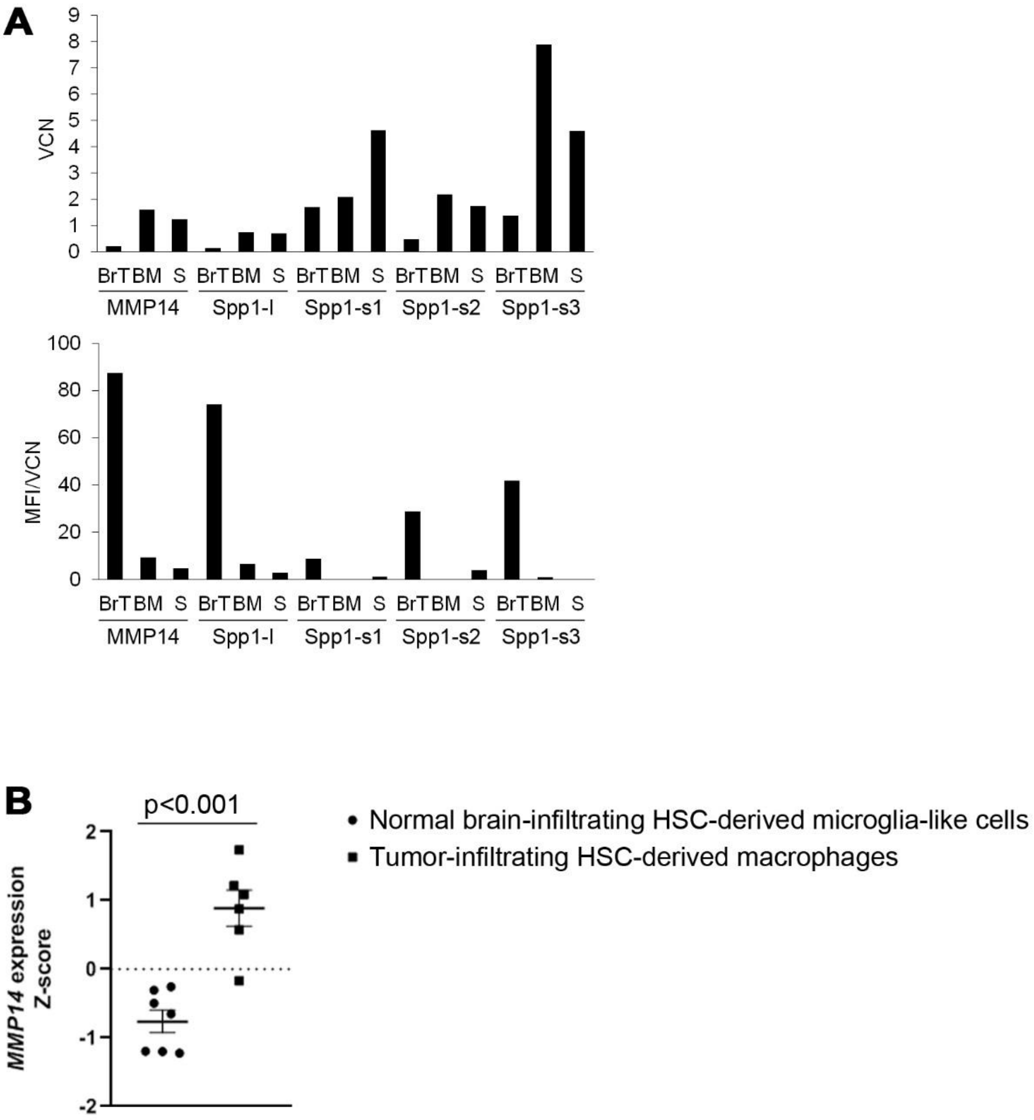

Supplementary Figure 8. Validation of promoter constructs in preclinical models and *Mmp14* expression in HSC-derived cells engrafting in experimental brain metastases and in normal brain. **(A)** Top graph: Vector copy number (VCN) analysis in gDNA isolated from brain tumors (BrT), bone marrow (BM) and spleens (S) of mice that have received HSCs transduced with MMP14:GFP, SPP1long:GFP (Spp1-l) or SPP1short:GFP (Spp1-s) constructs (MOI 20) prior to tumor establishment. VCN per genome is shown. Bottom graph: MFI normalized to VCN (MFI/VCN) for the same samples as shown in the top graph. Data for 5 individual mice are shown. **(B)** Publicly available gene expression data sets GSE86573, GSE122769 and GSE84819 were analyzed for expression of *Mmp14* gene, comparing HSC-derived cells infiltrating brain metastases versus normal brain.

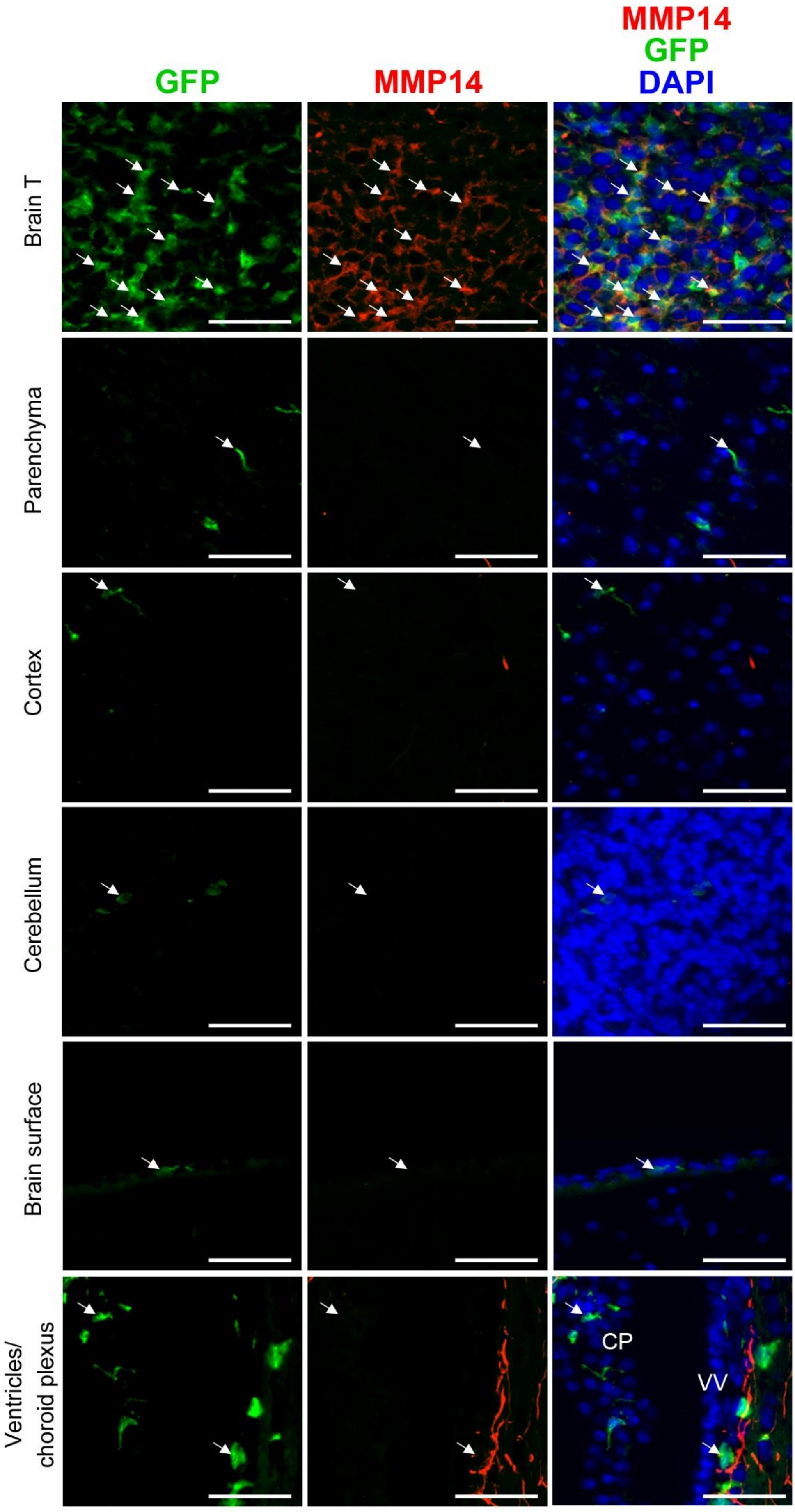
MMP14
GFP
DAPI
GFP
MMP14
Brain T
Parenchyma
Cortex
Cerebellum
Brain surface
Ventricles/
choroid plexus
CP
VV

Supplementary Figure 9. Expression of MMP14 in GFP+ cells within brain tumors and normal brain tissue. Images were taken in different brain area as indicated. Arrows point to representative GFP+ cells. CP, choroid plexus; VV, ventricle wall. Scale bars = 50 µm.

Supplementary Table 1. Percentage of GFP+ HSC-derived cells in different tissues and statistics analysis.

		**BrM**	**BM**	**Spleen**	**Blood**	**Lungs**
**MMP14:GFP**	% GFP+ cells*	3.7 (±4.1)	1.4 (±2.0)	0.6 (±0.7)	1.4 (±1.6)	6.3 (±3.6)
	p-value †	–	0.14	0.04	0.12	0.14
**SPP1short:GFP**	% GFP+ cells*	1.2 (±1.1)	0.1 (±0.1)	0.03 (±0.06)	0.1 (±0.1)	4.3 (±2.4)
	p-value †	–	0.22	0.21	0.24	0.11
**SPP1long:GFP**	% GFP+ cells*	2.4 (±2.1)	0.3 (±0.4)	0.1 (±0.1)	0.6 (±0.3)	2.5 (±1.3)
	p-value †	–	0.06	0.04	0.09	0.91

*Mean values (± standard deviation) for %GFP+ cells within CD45+ population infiltrating different organs is shown for tissues isolated from mice that have received HSCs transduced with promoter:reporter constructs expressing GFP under the Mmp14, Spp1long or Spp1 short promoter as indicated; BrM, Brain Metastases; BM, Bone Marrow.

† p values between BrM and the indicated tissue (two-tailed t-test; n=5).

Supplementary Table 2. Mean fluorescence intensity (MFI) of GFP in GFP+ HSC-derived cells infiltrating different tissues and statistics analysis.

		**BrM**	**BM**	**Spleen**	**Blood**	**Lungs**
**MMP14:GFP**	GFP MFI*	102.1 (±96.2)	22.8 (±22.6)	10.9 (±7.1)	22.4 (±17.5)	32.8 (±30.7)
	p-value †	–	0.002	0.002	0.002	0.016
**SPP1short:GFP**	GFP MFI*	35.1 (±18.9)	5.3 (±3.4)	0.000 (±0)	5.2 (±3.6)	12.8 (±3.9)
	p-value †	–	<0.001	<0.001	<0.001	<0.001
**SPP1long:GFP**	GFP MFI*	40.3 (±22.1)	5.6 (±1.4)	0.000 (±0.00)	5.0 (±2.7)	5.5 (±5.2)
	p-value †	–	<0.001	<0.001	<0.001	<0.001

*MFI of GFP signal (± standard deviation) for GFP+ cells within CD45+ population infiltrating different organs is shown for tissues isolated from mice that have received HSCs transduced with promoter:reporter constructs expressing GFP under the MMP14, Spp1long or Spp1 short promoter as indicated. BrM, Brain Metastases; BM, Bone Marrow.

† p values between BrM and the indicated tissue (two-tailed t-test; n=5)

Supplementary Table 3: Primers and conditions used for semiquantitative RT-PCR screening.

Gene	Forward primer	Reverse primer	Tm (°C)	Cycles	cDNA size (bp)
Spp1	GCTTGGCTTATGGACTGAGGTC	CCTTAGACTCACCGCTCTTCATG	53	30	114
Mmp14	GATGGACACAGAGAACTTCGTG	CGAGAGGTAGTTCTGGGTTGAG	53	30	117
Trem2	CTACCAGTGTCAGAGTCTCCGA	CCTCGAAACTCGATGACTCCTC	53	30	134
Dab2	CTCTTCAAAGGCAATGCTCCTGC	TATGGCTCCTGGGACCACAGTT	55	30	134
Emp1	TCCCTGTCCTACGGCAATGAAG	CTGGAACACGAAGACCACAAGG	60	35	169
Arg1	CATTGGCTTGCGAGACGTAGAC	GCTGAAGGTCTCTTCCATCACC	53	30	124
Ccl7	CAGAAGGATCACCAGTAGTCGG	ATAGCCTCCTCGACCCACTTCT	55	30	108
Cxcl10	ATCATCCCTGCGAGCCTATCCT	GACCTTTTTTGGCTAAACGCTTTC	55	32	134
Cxcl16	GCAGGGTACTTTGGATCACATCC	AGTTCACGGACCCACTGGTCTT	57	32	126
Ccr5	GTCTACTTTCTCTTCTGGACTCC	CCAAGAGTCTCTGTTGCCTGCA	54	35	131
Gapdh	GCACAGTCAAGGCCGAGAAT	GCCTTCTCCATGGTGGTGAA	54	40	151

Supplementary Table 4: Primers used for cloning of promoter-reporter constructs

Gene	Promoter region	Forward primer	Reverse primer	Tm (°C)	Cycles
Mmp14	-1968 to -83	GAGATTAATTAACAGATCTTGCTCTGGGATCATTTTGCC	GAGAACCGGTCCTTTGTCTTCTAAAGGAACGG	76	35
Spp1	-815 to -31	GAGATTAATTAACCTGTCACACACACATTGTAAAGTTC	GAGAACCGGTGAGGCACAGTTGATGTCTTTCAG	70	35
Spp1	-1475 to -31	GAGATTAATTAAGTAGTTAATGACATCGTTCATCAGTAATG CTTTGTG	GAGAACCGGTGAGGCACAGTTGATGTCTTGTCAG	74	35
Dab2	-998 to -1	GAGATTAATTAAGGATCTCATGGAGGCATTTCCCAAC	GAGAACCGGTGACGGGAAGGCAAGCAGAAAATCCAGC	75	35
